# Phytochemical synergies in BK002: advanced molecular docking insights for targeted prostate cancer therapy

**DOI:** 10.3389/fphar.2025.1504618

**Published:** 2025-02-17

**Authors:** Moon Nyeo Park, Jinwon Choi, Md. Maharub Hossain Fahim, Estéfani Alves Asevedo, Fahrul Nurkolis, Rosy Iara Maciel Azambuja Ribeiro, Han Na Kang, Sojin Kang, Rony Abdi Syahputra, Bonglee Kim

**Affiliations:** ^1^ Department of Pathology, College of Korean Medicine, Kyung Hee University, Seoul, Republic of Korea; ^2^ Experimental Pathology Laboratory, Midwest Campus, Federal University of São João del-Rei, Divinópolis, Brazil; ^3^ Department of Biological Sciences, State Islamic University of Sunan Kalijaga (UIN Sunan Kalijaga), Yogyakarta, Indonesia; ^4^ KM Convergence Research Division, Korea Institute of Oriental Medicine, Daejeon, Republic of Korea; ^5^ Korean Medicine-Based Drug Repositioning Cancer Research Center, College of Korean Medicine, Kyung Hee University, Seoul, Republic of Korea

**Keywords:** BK002, *Achyranthes japonica* (Miq.) Nakai, *Melandrium firmum* (Siebold and Zucc.) Rohrb, network pharmacology, molecular docking, 20-hydroxyecdysone, prostate cancer

## Abstract

*Achyranthes japonica* (Miq.) Nakai (AJN) and *Melandrium firmum* (Siebold and Zucc.) Rohrb. (MFR) are medicinal plants recognized for their bioactive phytochemicals, including ecdysteroids, anthraquinones, and flavonoids. This study investigates the anticancer properties of key constituents of these plants, focusing on the BK002 formulation, a novel combination of AJN and MFR. Specifically, the research employs advanced molecular docking and *in silico* analyses to assess the interactions of bioactive compounds ecdysterone, inokosterone, and 20-hydroxyecdysone (20-HE) with key prostate cancer-related network proteins, including 5α-reductase, CYP17, DNMT1, Dicer, PD-1, and PD-L1. Molecular docking techniques were applied to evaluate the binding affinities contributions of the bioactive compounds in BK002 against prostate cancer-hub network targets. The primary focus was on enzymes like 5α-reductase and CYP17, which are central to androgen biosynthesis, as well as on cancer-related proteins such as DNA methyltransferase 1 (DNMT1), Dicer, programmed death-1 (PD-1), and programmed death ligand-1 (PD-L1). Based on data from prostate cancer patients, key target networks were identified, followed by *in silico* analysis of the primary bioactive components of BK002.In silico assessments were conducted to evaluate the safety profiles of these compounds, providing insights into their therapeutic potential. The docking studies revealed that ecdysterone, inokosterone, and 20-hydroxyecdysonec demonstrated strong binding affinities to the critical prostate cancer-related enzymes 5α-reductase and CYP17, contributing to a potential reduction in androgenic activity. These compounds also exhibited significant inhibitory interactions with DNMT1, Dicer, PD-1, and PD-L1, suggesting a capacity to interfere with key oncogenic and immune evasion pathways. Ecdysterone, inokosterone, and 20-hydroxyecdysone have demonstrated the ability to target key oncogenic pathways, and their favorable binding affinity profiles further underscore their potential as novel therapeutic agents for prostate cancer. These findings provide a strong rationale for further preclinical and clinical investigations, supporting the integration of BK002 into therapeutic regimens aimed at modulating tumor progression and immune responses.

## 1 Introduction

### 1.1 Chronic inflammation and cancer: a complex interrelationship

The established link between chronic inflammation and cancer has illuminated the intricate biological processes that fuel carcinogenesis. Chronic inflammation, which can be driven by persistent infections, autoimmune disorders, or prolonged exposure to environmental carcinogens such as tobacco smoke, industrial pollutants, or asbestos, sets the stage for continuous tissue damage and abnormal cellular proliferation. This sustained inflammatory environment creates fertile ground for neoplastic transformations by promoting cellular repair mechanisms that, paradoxically, can lead to the onset of malignancies (Khandia and Munjal, 2020). Inflammation not only supports the survival and proliferation of cancer cells but also fosters metastasis (Hasegawa et al., 2006) by reshaping the tumor microenvironment (TME) to favor malignant growth (Afshari et al., 2022). Epigenetic alterations induced by inflammatory signaling further entrench the cancer-inflammation nexus, as oncogenes are activated and tumor suppressor genes are silenced through DNA methylation, histone modification, and chromatin remodeling (Tan et al., 2022). Within the TME, immune cells like macrophages, neutrophils, and T-cells play dual roles, either suppressing or facilitating tumor progression, thus complicating therapeutic interventions (Li et al., 2019; Segovia et al., 2019; Vredevoogd et al., 2019; Segovia et al., 2020).

A key mechanism by which tumors evade immune surveillance is through the upregulation of immune checkpoints such as programmed death-1 (PD-1) and programmed death ligand-1 (PD-L1), which inhibit T-cell activity and allow cancer cells to thrive unchecked (Wei et al., 2019). The successful targeting of immune checkpoints has revolutionized cancer treatment by reinvigorating the immune system’s ability to combat tumors, as evidenced by the clinical efficacy of anti-PD-1/PD-L1 and anti-CTLA-4 therapies (Jacquelot et al., 2019; Theivanthiran et al., 2020). Notably, the transcription factor NF-κB plays a pivotal role in regulating PD-L1 expression, directly binding to the promoter of the PD-L1 gene and upregulating its transcription. Additionally, NF-κB influences post-transcriptional pathways that stabilize PD-L1, contributing to tumor immune evasion (Antonangeli et al., 2020). Recent discoveries reveal that cancer stem-like cells (CSCs) exhibit heightened PD-L1 expression, further protecting them from immune attacks. The regulatory mechanisms that enrich PD-L1 expression in CSCs remain largely unexplored, underscoring a critical gap in our understanding of tumor resistance (Hsu et al., 2018).

Interestingly, the mesenchymal-epithelial transition (MET) has been identified as a novel mechanism regulating PD-L1 stability in CSCs, with studies showing that targeting this pathway can enhance the efficacy of cancer immunotherapy (Sharaf et al., 2014). The influence of chromatin remodeling in response to inflammatory signals has also gained attention, with genes like IL-1A and IL-1B becoming dynamically repositioned within transcription factories during immune responses. This spatial organization of gene expression is crucial for mediating inflammatory effects within tumors (Papantonis et al., 2012; Audia and Campbell, 2016; Horton et al., 2016; Marazzi et al., 2018; Shokri et al., 2018; Das et al., 2021; Ding et al., 2022). Similarly, the role of inflammasomes, particularly NLRP3, in cancer has been increasingly recognized, linking inflammation directly to cancer progression (Ding et al., 2022).

Despite significant advances in immunotherapies, including immune checkpoint inhibitors and CAR T-cell therapies, certain cancers, such as pancreatic and prostate cancers, have shown limited responses to these treatments (Ye et al., 2024). Understanding the molecular drivers of resistance, such as aberrant inflammatory signaling and immune evasion, remains key to improving the clinical outcomes of these malignancies. Combining immunotherapy with other treatment modalities, such as chemotherapy or radiotherapy, has shown promise, but additional research is required to fully unlock the potential of these therapeutic strategies (Latchman et al., 2001; De Marzo et al., 2007; Gandaglia et al., 2013; Powles et al., 2014). Furthermore, focusing on critical protein networks in cancer progression offers an opportunity to develop more precise, multi-targeted therapies that address the complexity of cancer’s molecular landscape. This approach holds the promise of better patient outcomes and a more profound understanding of the interplay between inflammation, the immune system, and cancer.

### 1.2 Comparative expression analysis of DNMT-1, Dicer1, and PD-1/PD-L1 in normal and prostate cancer patients and their correlation with gleason score

**FIGURE 1:**
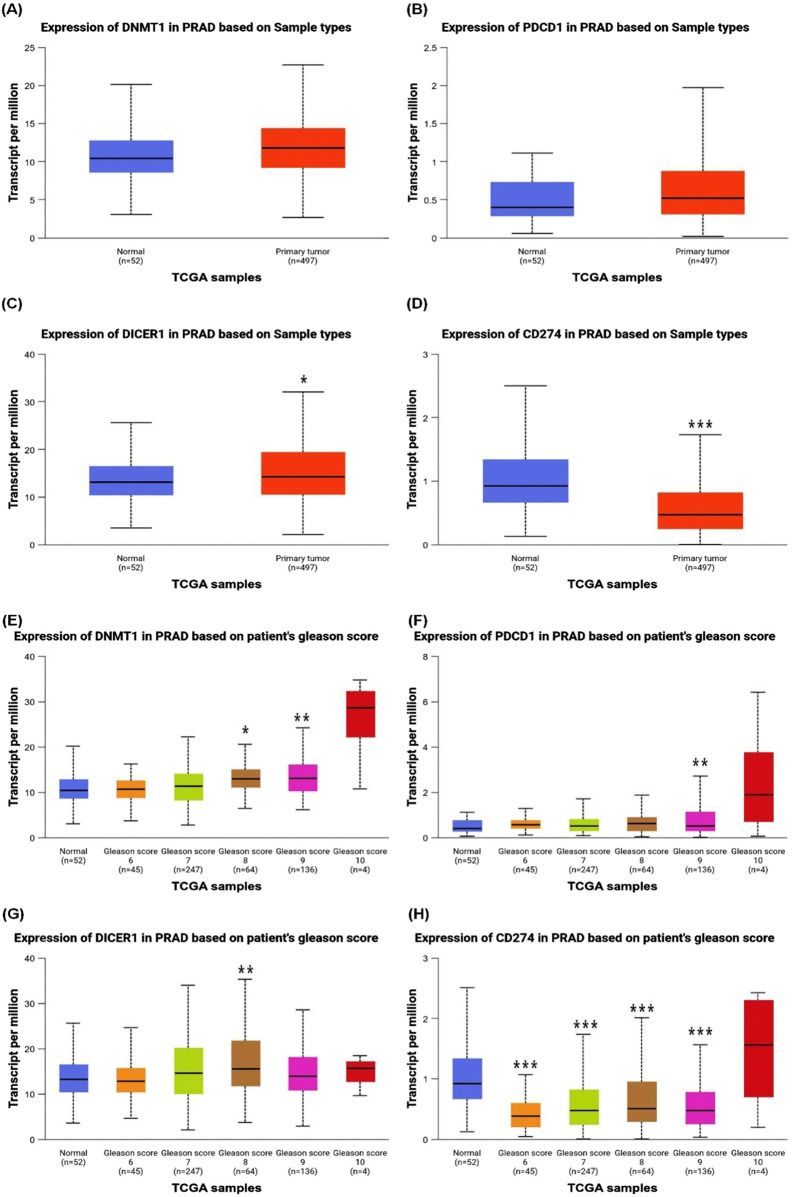
Differential Gene Expression Profiles in Prostate Cancer Patients. Graphical representation of the expression levels of DNMT-1, PD1, Dicer1, and PD-L1 in prostate cancer patients compared to normal individuals. The expression of **(A)** DNMT-1, **(B)** PD1, **(C)** Dicer1, and **(D)** PD-L1 is shown, highlighting significant differences between normal and cancerous tissues (p-values: p* < 0.05, *p* *** < 0.001, as indicated). Additionally, panels **(E)** DNMT-1, **(F)** PD1, **(G)** Dicer1, and **(H)** PD-L1 illustrate the correlation of their expression with the Gleason score, representing prostate cancer progression and aggressiveness. Statistical significance is indicated by corresponding p-values, demonstrating the relationship between molecular expression and tumor grade (p-values: p* < 0.05, p** < 0.01, *p* *** < 0.001, as shown).

Figure 1 provides a comprehensive analysis of the expression levels of specific genes DNMT-1, PD1, Dicer1, and PD-L1 in prostate cancer patients compared to normal individuals, sourced from https://ualcan.path.uab.edu/index.htm. These levels are correlated with a Gleason score, a grading system used to evaluate the aggressiveness of prostate cancer.

Figure 1A reveals that DNMT-1 expression is significantly elevated in prostate cancer patients compared to normal individuals. DNMT-1, a key enzyme involved in DNA methylation, plays a crucial role in maintaining the epigenetic landscape of cancer cells, contributing to tumor progression and silencing tumor suppressor genes (Park, 2023). Figure 1B describes the expression of PD1, a protein that negatively regulates T-cell activity, that is implicated in immune evasion by tumors, and that is notably higher in cancer patients than in normal individuals. This upregulation suggests that prostate cancer cells may employ immune checkpoint mechanisms to evade immune surveillance. In silico clinical trials, while offering valuable insights, must be interpreted with an understanding of their inherent limitations (Creemers et al., 2023). In our analysis, no statistically significant correlation was observed between DNMT-1 and PD-1 expression levels and patient outcomes. These findings highlight the importance of complementing *in silico* results with experimental validation and clinical studies to ensure robust and reliable conclusions (Figures 1A, B).

Figure 1C shows that Dicer1, an enzyme essential for microRNA processing (Goel and Goel, 2024), also exhibits increased expression in prostate cancer tissues compared to normal tissues. Elevated Dicer1 levels may contribute to the dysregulation of microRNA pathways that are critical in cancer cell proliferation, invasion, and metastasis (Dobrijević et al., 2021).

In normal cells, PD-L1(CD274) expression is typically low or tightly regulated, and its upregulation occurs primarily in response to inflammatory signals (e.g., cytokines like IFN-γ) or under conditions of immune activation (Sun H. Y et al., 2020). As shown Figure 1D, overall downregulation of PD-L1 expression in prostate cancer tissues compared to normal tissues. This suggests that, at the transcriptional level, PD-L1 expression may not universally increase in prostate cancer.

The expression dynamics of PD-L1 (CD274) in prostate cancer are complex and can vary depending on tumor stage, tumor microenvironment, and specific cellular contexts. While some studies report elevated PD-L1 levels in advanced or metastatic prostate cancer (Zavridou et al., 2021). Figure 1H indicates a positive correlation between PD-L1 expression and higher Gleason scores. This suggests that, while PD-L1 expression may be lower overall in prostate cancer tissues, higher-grade tumors may exhibit relatively elevated PD-L1 levels compared to lower-grade tumors within the prostate cancer cohort. PD-L1 binds to PD1 on T-cells, leading to the suppression of the immune response, which allows cancer cells to thrive by avoiding immune detection and destruction (Topalian et al., 2015; Chen and Mellman, 2017).

The correlation of gene expression with Gleason score is described in Figure lG−H. Figure 1E shows a positive correlation between DNMT-1 expression and the Gleason score, indicating that DNMT-1 expression increases with the severity and aggressiveness of prostate cancer. Figure lF reveals similar trend with PD1, where its expression levels rise in tandem with the Gleason score. This suggests that as prostate cancer becomes more aggressive, it increasingly relies on immune checkpoint pathways to evade the immune response. The expression of Dicer1 also shows a positive correlation with the Gleason score, as shown in Figure lG. This indicates that more aggressive prostate cancers may depend on the dysregulation of microRNA processing pathways, mediated by Dicer1, to sustain their growth and spread. Finally, Figure lH shows that PD-L1 expression also shows a strong positive correlation with the Gleason score, reinforcing the notion that more aggressive prostate cancers are more adept at suppressing immune responses, which is crucial for their survival and proliferation.

The data presented in this figure suggest that the expression of DNMT-1, PD1, Dicer1, and PD-L1 is markedly higher in prostate cancer patients compared to normal individuals and that their expression levels are positively correlated with the Gleason score. This indicates that these genes may play a critical role in the progression and aggressiveness of prostate cancer, and they could serve as potential biomarkers for disease severity or as targets for therapeutic intervention. The strong correlation between these gene expressions and the Gleason score underscores their potential utility in predicting the aggressiveness of prostate cancer and tailoring treatment strategies accordingly.

### 1.3 Prostate cancer pathology

Prostate cancer is the second most commonly diagnosed cancer worldwide and the fifth leading cause of cancer-related mortality among men as of 2022, with an estimated 1.5 million new cases and 397,000 deaths globally. Notably, in 52 countries, it stands as the leading cause of cancer-related deaths among men, underscoring its significant global health burden (Bray et al., 2024). Prostate cancer is a complex disease influenced by numerous biological, environmental, and genetic factors, with inflammation playing a significant role in its development and progression. The prostate is an immunocompetent organ, often the site of a small number of inflammatory cells (Gandaglia et al., 2013). Prostatic inflammation can stem from various sources, such as viral or bacterial infections, dietary factors, hormonal imbalances, autoimmune responses, and even urine reflux (De Marzo et al., 2007; Gandaglia et al., 2013). Accumulating evidence from epidemiological, histopathological, and molecular research strongly supports a link between chronic inflammation and the initiation and advancement of prostate cancer (De Nunzio et al., 2011; Sfanos et al., 2014). As inflammation persists, it creates a microenvironment that fosters cellular proliferation, DNA damage, and epigenetic changes, all of which contribute to malignant transformation and tumor progression.

A cornerstone of prostate cancer treatment, especially in cases of locally advanced or metastatic disease, has been androgen deprivation therapy (ADT), which can be administered either pharmacologically or surgically (El Badri et al., 2019). Androgen suppression has long been recognized as a vital strategy in controlling prostate cancer, dating back to 1941 when it was discovered that reducing testosterone levels could slow tumor growth (Huggins and Hodges, 1941). Historically, castration, along with the use of estrogen injections to inhibit testosterone production, proved effective in managing the disease (Mcleod, 2003; Sharifi et al., 2005). Over time, ADT evolved to offer more sophisticated approaches such as anti-androgens that block testosterone receptors on prostate cancer cells. Another widely used method involves luteinizing hormone-releasing hormone (LHRH) agonists that prevent the anterior pituitary gland from secreting luteinizing hormone, thereby reducing testosterone production (Litwin and Tan, 2017).

Emerging research has highlighted the intricate interplay between androgen signaling, growth hormone (GH), and estrogen pathways in prostate cancer. GH and gonadal systems are intimately linked in terms of growth, development, and metabolism, yet their precise regulatory interactions remain only partially understood (Marin et al., 1994). The enzyme aromatase, which converts androgens into estrogens in various tissues, has revealed a deep mechanistic connection between estrogen biology and GH regulation. Local estrogen production can exert paracrine effects that extend beyond classical endocrine pathways, influencing GH secretion even in the absence of the androgen receptor (Birzniece and Ho, 2021). Additionally, 5α-reductase, which converts testosterone into dihydrotestosterone (DHT), plays a crucial role in androgen action at the tissue level, further impacting GH regulation and prostate cancer progression (Veldhuis et al., 2009).

Testosterone acts as a prohormone, its effects being mediated through its conversion into DHT and estradiol (E2) in tissue-specific contexts (Jasuja et al., 2013; van den Beld et al., 2018; Kaufman et al., 2019). Research has demonstrated that inhibiting 5α-reductase, thereby reducing DHT, does not affect testosterone’s ability to increase GH secretion. However, inhibition of aromatase, which reduces estradiol levels, significantly disrupts GH production, revealing estradiol as a critical mediator of GH’s effects in prostate cancer (Link et al., 1986; Birzniece and Ho, 2021). In recent clinical studies, 5α-reductase inhibitors have been explored as a potential therapeutic option for prostate cancer patients (Hu et al., 2020). Finasteride and dutasteride, two synthetic 5α-reductase inhibitors (5ARIs), are commonly recommended for treating conditions such as alopecia, lower urinary tract symptoms, and benign prostatic hyperplasia. However, these medications carry a range of significant side effects, including a heightened risk of high-grade prostate cancer as well as neurological, psychiatric, endocrine, metabolic, and ophthalmological issues. Because 5ARIs are lipophilic, they can cross the blood-brain barrier, potentially disrupting neurosteroid synthesis, altering neurochemistry, and impairing neurogenesis. These effects underscore the urgent need for further research into the long-term impact of 5ARIs and for innovative therapeutic solutions (Leliefeld et al., 2023). The complexity of these hormonal pathways underscores the importance of understanding the broader regulatory networks that influence cancer progression.

Furthermore, the link between obesity and prostate cancer has become increasingly evident, as obesity triggers pathways related to insulin resistance, chronic inflammation, and oxidative stress. These pathways, including the IGF-1 system, adipokine signaling, and the distribution of sex hormones, create a favorable environment for cancer initiation and progression (Birzniece et al., 2010; Birzniece et al., 2019). Insulin and IGF-1, both key players in metabolic regulation, share sequence similarities and can activate oncogenic signaling pathways such as MAPKs and PI3K-AKT, which are known to promote cancer cell proliferation and inhibit apoptosis. The dysregulation of these pathways in obesity enhances the autocrine and paracrine promotion of cancer, further complicating treatment strategies (O’Brien et al., 2005; Fogarty et al., 2008; Pollak, 2008; Avgerinos et al., 2019). Hormone-related cancers, particularly those affecting organs governed by intricate feedback mechanisms, are profoundly impacted when autocrine pathways, such as insulin-related signaling, become dysregulated. The activation of these feedback loops can amplify oncogenic signals, driving cancer progression and complicating treatment strategies (Park et al., 2022). Addressing this challenge requires targeted therapeutic approaches that restore and maintain homeostasis within these systems while mitigating the risks associated with hormonal and metabolic imbalances.

In addition to hormonal and metabolic factors, bone health is a critical consideration in prostate cancer management, especially given the high incidence of bone metastasis in advanced stages of the disease. Sustained ADT significantly reduces bone mineral density (BMD), leading to an increased risk of fractures and osteoporosis (Zhang et al., 2020). Studies show that BMD can decrease by between 4% and 13% annually during ADT, with the risk of fractures rising correspondingly (Miyazawa et al., 2018). To address these complications, it is essential to implement comprehensive bone health evaluations before initiating ADT. Recent guidelines have begun to prioritize bone health in prostate cancer management, recommending the use of bone-modifying agents (BMAs), such as bisphosphonates and denosumab, to mitigate bone loss and reduce fracture risk (Chakhtoura et al., 2021; Khan, 2023). However, patients with metastatic castration-resistant prostate cancer (mCRPC) have not seen an improvement in overall survival (OS) with bisphosphonates or denosumab. Nonetheless, having demonstrated the ability to postpone or avoid skeletal-related events (SREs) in patients with mCRPC, both BMAs have been approved in this context (Saad et al., 2004; Fizazi et al., 2011). About 90%–95% of patients with hormone-sensitive prostate cancer (mHSPC) have bone metastases (Fizazi et al., 2017; Parker et al., 2018). Unlike mCRPC, however, there is little data to support prescription of bone protective medicines in mHSPC (Cattrini et al., 2019). Notably, the lack of effective bone-protective agents in managing mHSPC underscores the urgent need to explore and develop novel therapies tailored to support bone health in prostate cancer patients. This complexity further highlights the necessity for multi-target therapeutic strategies capable of addressing the diverse mechanisms driving prostate cancer progression. By targeting metabolic dysregulation, hormonal feedback imbalances, and bone health simultaneously, multi-component approaches hold the potential to significantly enhance treatment efficacy, improve clinical outcomes, and ultimately elevate the quality of life for patients.

Next-generation anti-androgens like apalutamide, darolutamide, and enzalutamide have shown higher efficacy compared to first-generation agents, not only by competitively inhibiting the AR ligand-binding domain but also by preventing AR translocation to the nucleus and AR-mediated transcription (Crawford et al., 2018).

One of the most significant challenges in treating prostate cancer is the emergence of resistance to androgen receptor (AR)-targeted therapies. A major contributor to this resistance is the androgen receptor splice variant 7 (AR-V7), which lacks the ligand-binding domain necessary for traditional AR inhibitors to be effective (Antonarakis et al., 2016). AR-V7 remains constitutively active, driving cancer progression even in the absence of androgens. This splice variant has been detected in circulating tumor cells (CTCs) and is associated with resistance to androgen receptor axis-targeted agents (ARATs) such as enzalutamide and abiraterone (Antonarakis et al., 2016; Scher et al., 2016). Only a few of the more than 20 distinct AR variations that have been found have undergone in-depth research. The most extensively studied AR variations, outside AR-V7, are AR-V1, AR-V3, AR-V9, and ARv567es (Armstrong and Gao, 2019). For patients who express AR-V7, chemotherapy may prove more effective than AR-directed therapy, and ARATs combined with ADT remain standard treatment options for castration-resistant prostate cancer (CRPC) (Scher et al., 2012; Fizazi et al., 2014; Ryan et al., 2015; Hussain et al., 2018; Armstrong et al., 2019; Davis et al., 2019; Fizazi et al., 2019; Sternberg et al., 2020). The concurrent use of abiraterone acetate with low-dose prednisone is crucial to mitigate mineralocorticoid-related side effects, such as hypertension and fluid retention, further illustrating the complexity of managing advanced prostate cancer (Hatano and Nonomura, 2023).

Thus, therapeutic resistance remains a significant challenge in targeted cancer treatment due to tumor cell plasticity, which drives the emergence of resistance mechanisms such as target mutations, pathway reactivation, and interactions with the tumor microenvironment. Although targeted therapies hold great potential for personalized cancer treatment, the adaptability of tumor cells and their inherent heterogeneity often complicate treatment responses. However, a deeper understanding of these resistance mechanisms has led to the development of combination therapies, which are showing promise in improving therapeutic outcomes by overcoming resistance and enhancing treatment efficacy (Ramos and Bentires-Alj, 2015).

Innovative therapeutic strategies continue to emerge, including the exploration of tyrosine kinase inhibitors, vaccination therapies, immune checkpoint inhibitors (such as PD-1/PD-L1 and CTLA-4 inhibitors), PARP inhibitors, and PSMA-targeted treatments (Mitsogiannis et al., 2022). The heterogeneity of prostate cancer, particularly the plasticity induced by ADT, highlights the need for early multi-modal therapy that targets diverse mechanisms to prevent resistance and improve patient outcomes (Fizazi et al., 2017; James et al., 2017; Gravis et al., 2018; Kyriakopoulos et al., 2018). Prostate cancer patient data and overall disease progression indicate the significance of focusing on the network that comprises the main drivers. Thus, it is becoming clear that a more logical approach based on a more holistic strategy is to target the larger cancer-causing networks rather than to depend on single-target therapy. This approach attempts to interfere with the several processes that lead to tumor growth and resistance, acknowledging the complex nature of prostate cancer.

## 2 Challenges for targeting multiple pathways in prostate cancer therapeutics

### 2.1 The role of DNMT1 in prostate cancer progression

The rapid demethylation of 5-methylcytosine (5 mC) during epigenetic reprogramming, particularly during cancer progression, cannot be entirely explained by passive methyl loss during replication. Active enzymatic processes also play a crucial role in this demethylation, contributing to significant alterations in gene expression that fuel tumorigenesis (Wu and Zhang, 2010). Intratumoral heterogeneity (ITH) is increasingly recognized as a product of aberrant CpG methylation, which disrupts alternative splicing mechanisms and contributes to cancer’s adaptive and aggressive behavior (Lin et al., 2023). The interplay between genetic mutations and epigenetic modifications, such as DNA methylation and histone modifications, is a fundamental driver of oncogenesis (Network et al., 2013; Bullinger et al., 2017; Eisfeld et al., 2020), impacting key oncogenes and tumor suppressor genes to foster tumor progression and metastasis (Aguilera et al., 2010).

This detailed discussion brings to light how DNA methyltransferases (DNMTs), particularly DNMT1, are integral to this process (Elenbaas et al., 2001; Taube et al., 2013). For instance, DNMT1’s role in the epithelial-mesenchymal transition (EMT) and cancer stem cell (CSC) phenotypes highlights its significant impact on tumor initiation and progression (Lee et al., 2016). The co-expression of DNMT1 and the Enhancer of zeste homolog 2 (EZH2), alongside their correlation with poor prognostic markers in prostate cancer, underscores the importance of DNMT1 in maintaining tumor-promoting epigenetic landscapes (Li et al., 2022). Additionally, mechanisms involving other key proteins, such as calcium/calmodulin-dependent protein kinase II inhibitor I (CAMK2N1), which appears to be downregulated via promoter hypermethylation, further emphasize the role of DNMT1 in cancer (Peng W et al., 2023). This has therapeutic implications, as targeting DNMT1-mediated methylation could reactivate tumor suppressor genes like FAM107A, which is silenced in PCa through CpG island hypermethylation. FAM107A acts as a molecular brake on the FAK/PI3K/AKT pathway, and its reactivation could inhibit tumor growth and metastasis, offering a potential therapeutic target (Ke et al., 2022). Moreover, miRNAs, such as miRNA-148a and miRNA-125b, regulate DNMT1 and p53, influencing gene silencing and TP53-related pathways.

The therapeutic manipulation of these miRNAs in PCa could modulate DNMT1 activity and reverse oncogenic methylation patterns (Melnik, 2017). For example, mahanine, a plant-derived alkaloid, restores tumor suppressor gene expression by inhibiting DNMT1 degradation, revealing the therapeutic potential of natural compounds in targeting epigenetic machinery (Agarwal et al., 2013). Thus, the role of DNMT1 in the modulation of enhancer RNA (eRNA) linked to the androgen receptor (AR) suggests that targeting DNMT1 in these non-coding RNA interactions could provide new avenues for diagnostics and therapy. This complexity highlights the profound influence of epigenetic regulation in cancer biology, with DNMT1 as a central figure in the maintenance of oncogenic states (Pan et al., 2021). Nucleoside analogs cause DNA double-strand breaks and cell death, in addition to depleting DNMTs and lowering DNA methylation levels [12]. By reactivating endogenous retroviral elements, they also promote immunological responses via the viral defense pathway [21]. In prostate cancer, intratumoral heterogeneity (ITH) plays a critical role in driving local recurrence following radiation therapy. Emerging research highlights that the ecological interactions among distinct tumor cell subpopulations may significantly contribute to treatment resistance. This study aims to evaluate the impact of these intercellular dynamics on prostate cancer progression and their influence on the therapeutic response to radiation, providing new insights into the complexity of treatment resistance mechanisms (Paczkowski et al., 2021). Prostate cancer may exhibit reduced aggressiveness upon termination of DNMT1 expression, as this leads to a corresponding decrease in Enhancer of Zeste Homologue (EZH2) expression. DNMT1 promotes prostate cancer progression and metastasis by enhancing TRAF6 transcription and facilitating TNF receptor-associated factor 6 (TRAF6)-mediated ubiquitination of EZH2, underscoring its pivotal role in tumorigenesis and potential as a therapeutic target (Li et al., 2022). The development of epithelial-to-mesenchymal transition (EMT) and cancer stem cell (CSC) phenotypes within tumors is closely tied to the epigenetic regulation of genetic programs by DNA methyltransferases (DNMTs). In prostate cancer, EMT-driven bone metastasis is further facilitated by cancer-associated fibroblasts (CAFs), which enhance stromal CXC motif chemokine 12 (CXCL12) levels, creating a microenvironment that supports metastatic progression (Lee et al., 2016). The tumor suppressor gene calcium/calmodulin-dependent protein kinase II inhibitor I (CAMK2N1) is significantly downregulated in prostate cancer. This downregulation is driven by DNMT1-mediated DNA methylation, which not only suppresses CAMK2N1 expression but also triggers activation of the AKT and ERK signaling pathways. This activation establishes a feedback loop that promotes further DNMT1 production, amplifying oncogenic signaling and tumor progression (Peng Y et al., 2023). The FAM107A gene, located on the short arm of chromosome 3, is frequently downregulated in prostate cancer and is associated with poor prognosis. This downregulation is primarily driven by hypermethylation of CpG islands in its promoter region. Notably, overexpression of FAM107A has been shown to inhibit tumor cell motility, invasion, and proliferation while promoting apoptosis, primarily through modulation of the focal adhesion kinase (FAK)/PI3K/AKT signaling pathway (Ke et al., 2022). Milk-derived miRNAs, particularly miRNA-125b and miRNA-148a, influence the p53-DNMT1 regulatory axis, which governs key genes like BIRC5 (Baculoviral IAP Repeat Containing 5) (surviving) involved in cell survival and tumor progression. miRNA-125b targets TP53, altering p53-dependent gene networks, while miRNA-148a directly downregulates DNMT1, affecting chromatin regulation via Histone Deacetylase 1 (HDAC1). This milk-mediated miRNA-p53-DNMT1 pathway may explain the epidemiological link between milk consumption, acne vulgaris, and prostate cancer (Melnik, 2017). DNMT1 knockdown reduces repressive histone marks, particularly H3K9me3 and H3K27me3, on the promoters of Zinc Finger E-Box Binding Homeobox 2 (ZEB2) and Kruppel-like transcription factor 4 (KLF4) genes crucial for maintaining the EMT and CSC phenotype. This epigenetic alteration facilitates the transcriptional activation of these genes, driving aggressive tumor behavior and underscoring DNMT1’s pivotal role in modulating epigenetic landscapes within PCa cells (Lee et al., 2016). The multifaceted impact of DNMT1 across various signaling pathways and cellular mechanisms underlines its potential as a critical target in future prostate cancer treatments, particularly in strategies aimed at overcoming metastasis and resistance to conventional therapies. Table 1 summarizes the key points related to DNMT1’s role in prostate cancer.

**TABLE 1:** The complex role of DNMT-1 in Prostate cancer.

Key points	Mechanism	References
5 mC Loss	Rapid loss of 5 mC during cancer progression, involving active enzymatic processes, leading to significant gene expression alterations and tumorigenesis in prostate cancer patient	(Storebjerg et al., 2018)
ITH	ITH contributes to local recurrence in prostate cancer following radiation therapy, and interactions among diverse tumor cell subpopulations may drive treatment resistance in PC3 and DU145 cells	Paczkowski et al. (2021)
DNMT1 and EZH2 Co-Expression	Co-expression linked with poor prognostic markers in prostate cancer, maintaining tumor-promoting epigenetic landscapes in WPMY-1, DU145, PC-3, PC-3-shCtrl/shDNMT1#1, and WPMY-1-Vector/DNMT1/TRAF6/(DNMT1+TRAF6) cells	Li et al. (2022)
EMT	DNMT1 drives EMT, promoting increased invasiveness. It also facilitates the transition to a CSC phenotype, enhancing tumor progression and resistance to therapy in PC3 and DU145 cells	(Lee et al., 2016)
CAMK2N1	Downregulated via promoter hypermethylation, emphasizing DNMT1’s role in cancer progression	Peng Y et al. (2023)
FAN107A	DNMT1’s role in silencing FAM107A contribution to tumor progression through the FAK/PI3K/AKT signaling pathway	Ke et al. (2022)
Metastasis	DNMT1 knockdown enhances EMT induction and promotes the CSC phenotype. This reduction also decreases H3K9me3 and H3K27me3 levels on the Zeb2 and KLF4 promoters, as revealed in PC3^GFP^ and DU145^GFP^ cells	Lee et al. (2016)
miRNA-125b miRNA-148a	miRNA-125b and miRNA-148a, modulate the p53-DNMT1 pathway, influencing genes like BIRC5 (survivin) involved in prostate cancer (human and bovine)	Melnik (2017)

Abbreviation: 5-methylcytosine (5 mC): Intertumoral heterogeneity (ITH): DNA, methyltransferase (DNMT); Enhancer of Zeste Homologue (EZH2): TNF, receptor-associated factor 6 (TRAF6): Epithelial-to-mesenchymal transition (EMT): Cancer stem cell (CSC): Calcium/calmodulin-dependentprotein kinase II, inhibitor I (CAMK2N1): Focal adhesion kinase (FAK): Baculoviral IAP, Repeat Containing 5 (BIRC5): Zinc Finger E-Box Binding Homeobox 2 (ZEB2): Kruppel-like transcription factor 4 (KLF4).

### 2.2 The role of dicer in prostate cancer progression

Dicer, an essential enzyme in the biogenesis of microRNAs (miRNAs), plays a crucial role in prostate cancer progression by regulating various cellular processes, including cell division, apoptosis, and tumor invasion. In prostate cancer, Dicer expression is notably elevated in cancerous tissues compared to benign counterparts, particularly in early-stage disease, with higher levels correlating to more aggressive phenotypes. Elevated Dicer and Ago2 expression in prostate cancer tissues compared to adjacent benign tissues have been linked to lower Gleason scores, suggesting a role in moderating tumor aggression. When Dicer or Ago2 expression is silenced *in vitro*, prostate cancer cell lines such as LNCaP, PC-3, and DU145 exhibit significant reductions in cell proliferation and increased cell death, indicating Dicer’s role in promoting tumor cell survival. Moreover, Dicer inhibition leads to cell cycle arrest in the G2/M phase in androgen-dependent LNCaP cells and in the S phase in androgen-independent PC-3 and DU145 cells, demonstrating its influence on cell cycle regulation across different prostate cancer subtypes (Bian et al., 2014).

Dicer dysfunction is observed across various cancer types, including prostate cancer, where altered miRNA processing contributes to disease progression. Dicer’s role in miRNA maturation enables miRNAs like miR-200a and miR-31, which are downregulated in prostate cancer tissues, to serve as potential diagnostic and prognostic markers. Interestingly, metastatic prostate cancer exhibits elevated expression levels of miR-200a, miR-370, and miR-31 compared to localized prostate cancer, suggesting Dicer’s differential regulation in advanced disease stages (Bian et al., 2015). Transforming growth factor-β1 (TGF-β1) plays a central role in regulating EMT through its influence on nc886, a non-coding RNA transcribed by RNA polymerase III (Pol III). nc886 affects EMT indirectly by modulating the processing of microRNAs via Dicer, an essential enzyme in RNA silencing. Additionally, TGF-β1 regulates MYC-associated zinc finger protein (MAZ), a transcription factor that suppresses TGFBI, a gene involved in cell adhesion and migration. This regulatory framework reveals a novel EMT unit comprising nc886 and its neighboring genes, driven by TGF-β1-mediated differential transcription of Pol II and Pol III genes. Understanding this network provides new insights into EMT regulation in PCa and highlights potential therapeutic targets for mitigating metastasis (Yang et al., 2022).

Disruption of Dicer function has been shown to increase apoptosis and senescence in prostate cancer cell models, driven by upregulation of tumor suppressors such as P16/INK4a and P27/Kip1. This suggests that Dicer acts as a survival factor in prostate cancer cells, contributing to the maintenance of tumor growth and resistance to apoptosis in PrEC cells, PNT1a and PNT2, LNCaP, PC-3, DU145, and CWR22Rv1 cells (Zhang et al., 2014). Immunohistochemical studies on prostate cancer tissues have demonstrated Dicer overexpression in prostatic intraepithelial neoplasia (PIN) and in over 80% of prostate adenocarcinomas, indicating its potential role as a biomarker for early detection and progression monitoring (Chiosea et al., 2006). Furthermore, hypoxic conditions, often present in the tumor microenvironment, exacerbate Dicer dysfunction, leading to the downregulation of critical miRNAs like miR-124 and miR-144, which are associated with autophagy and treatment resistance. Overexpression of these miRNAs in hypoxic conditions has been shown to enhance radiosensitivity by downregulating PIM1, a key factor in prostate cancer progression (Gu et al., 2016).

In addition to its role in miRNA biogenesis, Dicer is involved in androgen receptor (AR) reprogramming, particularly in the transition to CRPC. Overexpression of MIR222HGs promotes androgen-independent growth in HSPC LNCaP cells by suppressing androgen receptor activity and reducing the expression of key AR-regulated genes (KLK3, TMPRSS2, FKBP5), driving the transition toward a CRPC phenotype. This suggests that targeting Dicer and its associated miRNAs could provide novel therapeutic avenues for combating CRPC (Sun et al., 2018).

Here, Dicer is a pivotal regulator of prostate cancer progression, influencing key processes such as cell proliferation, apoptosis, miRNA biogenesis, and androgen receptor signaling. Its role in regulating miRNAs and involvement in epigenetic modifications highlights its potential as a therapeutic target and biomarker in prostate cancer. Further investigation into Dicer’s mechanisms could uncover new strategies for targeted therapies, particularly in advanced and treatment-resistant forms of prostate cancer. Table 2 concisely summarizes the intricate role of Dicer in prostate cancer, highlighting its potential therapeutic and diagnostic applications.

**TABLE 2:** The intricate role of Dicer in Prostate cancer.

Key points	Mechanism	References
Cell Proliferation and Survival	Dicer silencing in prostate cancer cell lines (LNCaP, PC-3, DU145) reduces cell proliferation and increases apoptosis. It also leads to cell cycle arrest G2/M in LNCaP; S phase in PC-3	Liu et al. (2020)
miRNA Processing and Disease Progression	Dicer facilitates miRNA maturation. MiRNAs like miR-200a, miR-370, and miR-31 are elevated in metastatic prostate cancer, while miR-200a and miR-31 are downregulated in localized in PC-3, LNCaP	Bian et al. (2015)
EMT	EMT unit comprising nc886, modulated via Dicer, and its neighboring genes, is driven by TGF-β1-mediated differential transcription of Pol II and Pol III genes	Yang et al. (2022)
Impact on Apoptosis and Tumor Suppressors	Dicer dysfunction increases apoptosis and senescence in prostate cancer cells, involving upregulation of tumor suppressors such as P16/INK4a and P27/Kip1	Zhang et al. (2014)
Hypoxia-Induced Dicer Dysfunction	Hypoxic conditions reduce miRNAs like miR-124 and miR-144, leading to autophagy and treatment resistance. Overexpression of these miRNAs increases radio sensitivity by downregulating PIM1	Gu et al. (2016)
Androgen Receptor Reprogramming	Dicer regulates miRNA processing related to AR signaling, with overexpression of MIR222HG promoting androgen-independent tumor growth and altering AR-regulated genes	Sun et al. (2018)
Therapeutic Implications	Targeting Dicer and its associated miRNAs presents new therapeutic avenues for CRPC, especially in advanced, treatment-resistant cases	Ferreira et al. (2024)

Abbreviation: Transforming growth factor-β1 (TGF-β1): RNA, polymerase III (Pol III): Serine/threonine-protein kinase pim-1(PIM): Androgen receptor (AR): Castration-resistant prostate cancer (CRPC).

### 2.3 PD-1/PD-L1 pathway in prostate cancer

The PD-1/PD-L1 pathway plays a critical role in immune evasion mechanisms employed by prostate cancer cells, making it a pivotal target in the development of immunotherapeutic strategies (Gerger et al., 2011). PD-L1, a transmembrane protein encoded by the *CD274* gene, interacts with its receptor PD-1 on T cells, leading to the inhibition of T-cell activation and induction of T-cell anergy. This immune suppression enables tumor cells to evade immune detection and destruction. While targeting the PD-1/PD-L1 checkpoint has shown promise in a variety of cancers, including renal cell carcinoma, melanoma, and non-small cell lung cancer, prostate cancer, presents unique challenges due to its immunologically “cold” tumor microenvironment (Cha et al., 2019).

Collagen triple helix repeat containing 1 (CTHRC1) is associated with tumor progression and reduced disease-free survival in prostate cancer. High CTHRC1 expression correlates with increased levels of immune checkpoints PD-1 and PD-L1, enhanced infiltration of immune cells such as B cells, CD4⁺ T cells, macrophages, neutrophils, and dendritic cells, and is associated with genes like MMP9, MUC1, and SLC2B1 that drive PC progression. These findings suggest that CTHRC1 upregulation adversely affects PC prognosis and immune function, suggesting that targeting CTHRC1 could modulate the tumor microenvironment and improve therapeutic outcomes (Zhou et al., 2019).

Additionally, inhibition of heterogeneous nuclear protein L (HnRNP L) in castration-resistant prostate cancer (CRPC) reduces PD-L1 expression and destabilizes YY1 mRNA, thereby enhancing T-cell-mediated ferroptosis and improving antitumor immunity. This effect involves key molecules such as SLC7A11, Glutathione peroxidase 4 (GPX4), Signal transducer and activator of transcription 1(STAT1), Interferon gamma (IFN-γ), and Interleukin-1 (IL-2) (Zhou et al., 2022). Furthermore, PD-L1 protein levels in prostate cancer are regulated by proteasome-mediated degradation via Cyclin D-CDK4 and the Cullin 3^SPOP^ E3 ligase pathway. Blocking CDK4/6 with inhibitors increases PD-L1 levels, enhancing immune suppression, which suggest that combining CDK4/6 inhibitors with PD-1/PD-L1 immunotherapies could disrupt tumor immune evasion and improve patient outcomes (Zhang et al., 2018).

Prostate cancer’s low presence of tumor-infiltrating lymphocytes (TILs) contributes to immunotherapies targeting immune checkpoints, such as anti-PD-1 or anti-PD-L1 antibodies (Sharma and Allison, 2015; Gao et al., 2017; Subudhi et al., 2020). EP4 (PTGER4), expressed in epithelial and immune cells, modulates the prostate cancer immune microenvironment. YY001, a novel EP4 antagonist, inhibits the differentiation and immunosuppressive function of myeloid-derived suppressor cells (MDSCs) while enhancing T-cell proliferation and anticancer activity. This agent reverses MDSC and T-cell infiltration by altering tumor chemokine profiles, leading to increased CD8^+^ T-cell activation and reduced immunosuppressive functions (Peng et al., 2022).

Clinical studies have shown that docetaxel-based chemohormonal therapy increases tumor-infiltrating T cells by activating the cGAS/STING pathway and inducing IFN signaling. In xenograft mouse models, this therapy enhances T-cell infiltration and upregulates PD1/PD-L1 expression, sensitizing tumors to anti-PD1 blockade. A retrospective analysis of metastatic castration-resistant prostate cancer (mCRPC) patients demonstrated improved PSA progression-free survival with combined docetaxel and anti-PD1 therapy compared to anti-PD1 alone (Ma et al., 2022).

In metastatic castration-resistant prostate cancer (mCRPC), single-agent PD-1/PD-L1 inhibitors have shown limited efficacy, underscoring the need to “heat up” these tumors by enhancing immune cell infiltration (Huang and He, 2020). Strategies to convert “cold” tumors into “hot” tumors (those with increased T-cell infiltration and immune activity) are being actively investigated. One promising approach is the combination of PD-1/PD-L1 inhibition with therapies that target other immune-modulatory pathways, such as CXCR4, poly (ADP-ribose) polymerase (PARP), or transforming growth factor (TGF)-β inhibitors. Combining these therapies with immune checkpoint inhibitors has shown potential in enhancing antitumor responses and overcoming immune resistance (Majidpoor and Mortezaee, 2021).

Additionally, integrating PD-1/PD-L1 inhibitors with chemotherapy, radiation therapy, or other targeted therapies offer new opportunities for prostate cancer treatment. For instance, docetaxel (DTX) induce ATM-NF-κB signaling, upregulating PD-L1 expression and contributing to immune suppression. Combining DTX with PD-1/PD-L1 inhibitors may counteract this effect and restore immune system activity for more effective tumor control (Wang et al., 2021).

Using the Myc-CaP:PSMA (+) murine prostate cancer model and second-generation anti-hPSMA CAR T cells with a Click Beetle Red luciferase reporter, researchers evaluated CAR T cell trafficking and antitumor efficacy both alone and in combination with anti-PD-1 antibodies. They found that combining CAR T cell therapy with PD-1 blockade reversed the exclusion of CD3^+^ T cells from tumor centers and enhanced tumor treatment response, although the effect was short-lived. Additionally, an inverse pattern of CAR T cell bioluminescence was observed in treated tumors, linked to decreased mitochondrial function following T cell activation, highlighting metabolic challenges in solid tumor therapies (Serganova et al., 2017).

Clinical trials continue to explore the benefits of combining PD-1/PD-L1 inhibition with other therapeutic agents, such as tyrosine kinase inhibitors, PARP inhibitors, or radiotherapy, to enhance immune responses and improve clinical outcomes. Understanding the intricate regulation of PD-L1 expression and the immune microenvironment in prostate cancer is crucial to developing more effective and personalized treatment strategies (Rekoske et al., 2016). Although the “cold” immune environment of prostate cancer limits the effectiveness of PD-1/PD-L1 inhibitors as monotherapy, combination therapies hold significant potential. Ongoing research aims to uncover the mechanisms driving immune evasion in prostate cancer and develop novel therapeutic strategies that enhance immune cell infiltration and activity, ultimately improving patient outcomes in this challenging disease (Sharma et al., 2020).

To consolidate advances in immunotherapy, it is crucial to identify candidate agents that not only enhance our immune system but also maintain an overall balance. Such agents could transform the immune environment, potentially converting immunologically “cold” tumors into “hot” ones, thereby increasing the effectiveness of immune responses against cancer. Table 3 highlights the major points discussed in the text, providing an overview of the challenges, mechanisms, and potential strategies related to PD-1/PD-L1 immunotherapy in prostate cancer.

**TABLE 3:** The potential stages related to PD-1/PD-L1 immunotherapy in Prostate cancer.

Key points	Mechanism	References
CTHRC1	CTHRC1 is positively correlated with MMP9, MUC1, and SLC2B1.CTHRC1 may facilitate immune evasion by enhancing PD-1/PD-L1 signaling, leading to T cell exhaustion and reduced immune response	Zhou et al. (2019)
EP4/YY001	EP4 (PTGER4) modulates the prostate cancer immune microenvironment. YY001, an EP4 antagonist, inhibits MDSC differentiation and function while enhancing T-cell proliferation and antitumor activity. It reduces MDSC infiltration and boosts CD8^+^ T-cell activation, resulting in a robust antitumor immune response in clinical	Peng et al. (2022)
cGAS/STING, IFN	Docetaxel-based chemohormonal therapy increased tumor-infiltrating T cells by activating the cGAS/STING pathway and IFN signaling, leading to upregulation of PD1/PD-L1 expression and improved PSA progression-free survival in clinical trials	Ma et al. (2022)
HnRNP L, YY1	Inhibition of HnRNP L reduces PD-L1 expression and destabilizes YY1, leading to decreased levels of SLC7A11 and GPX4. This enhances T-cell-mediated ferroptosis and antitumor immunity by involving key factors such as STAT1, IFN-γ, IL-2, CD3, and CD28. These effects were observed both *in vitro* using co-cultures of PC3, DU145, RM-1, and Jurkat cells and *in vivo* in C57BL/6 mice	Zhou et al. (2022)
Regulatory and memory T lymphocytes	TIM-3 inhibition enhances anti-tumor immune responses in prostate cancer by counteracting the immunosuppressive effects of regulatory T cells (Tregs), thereby restoring T cell activity against prostate cancer	Molina et al. (2024)
Combining CDK4/6 inhibitor with PD-L1	The regulation of PD-L1 levels is controlled by proteasome-mediated degradation, influenced by CDK4/6 and Cullin 3^SPOP^ E3 ligase, where blocking the phosphorylation of SPOP can increase PD-L1 expression in primary prostate cancer specimens	Zhang et al. (2018), Palicelli et al. (2021)
Combining PD-1/PD-L1 with PARP inhibitors	The improvement of clinical outcomes may result from combining PD-1/PD-L1 inhibitors with tyrosine kinase inhibitors, PARP inhibitors, or radiotherapy	Mitsogiannis et al. (2022)
Combining PD-1/PD-L1, inhibitors with Other Therapeutic Agents	The combination of PD-1/PD-L1 inhibitors with CXCR4, PARP inhibitors, TGF-β inhibitors, chemotherapy, or radiation enhance in prostate cancer	Majidpoor and Mortezaee (2021), Palicelli et al. (2021)
Docetaxel and Immune Suppression	The upregulation of PD-L1 through ATM-NF-κB signaling by Docetaxel (DTX); combining DTX with PD-1/PD-L1 inhibitors may counteract this effect	Xie et al. (2018), Wang et al. (2021), Wu et al. (2023)
Ongoing Research Focus	The research focus is on understanding the mechanisms of immune evasion and developing strategies to enhance immune cell infiltration in prostate cancer	Wu et al. (2020)
Future Direction	The materials that enhance immune response and balance aid in converting “cold” tumors into “hot” tumors, thereby improving the effectiveness of immunotherapy	Mortezaee (2020a), Mortezaee, 2020b; Majidpoor and Mortezaee (2021)

Abbreviation: Programmed death receptor-1 (PD-1): PTGER4 (EP4): myeloid-derived suppressor cells (MDSCs): Heterogeneous nuclear protein L (HnRNP L): Signal transducer and activator of transcription 1 (STAT1): Interferon gamma (IFN-γ): Interleukin-1 (IL-2): speckle-type POZ, protein (SPOP): Glutathione peroxidase 4 (GPX4): Collagen triple helix repeat containing 1 (CTHRC1): matrix metalloproteinase-9 (MMP9): solute carrier organic anion transporter family member 2B1 (SLC2B1): Docetaxel (DTX): C-X-C chemokine receptor type 4 (CXCR-4): Poly (ADP-ribose) polymerase (PARP): Ataxia-telangiectasia mutated (ATM): Nuclear factor kappa-light-chain-enhancer of activated B cells (NF-κB): Chimeric antigen receptor (CAR) T-cell.

## 3 A promising herbal medicine

### 3.1 BK002 bioinformatics and network pharmacology analysis targeting DNMT1, dicer, PD-L1, and PD-1 in prostate cancer

**FIGURE 2:**
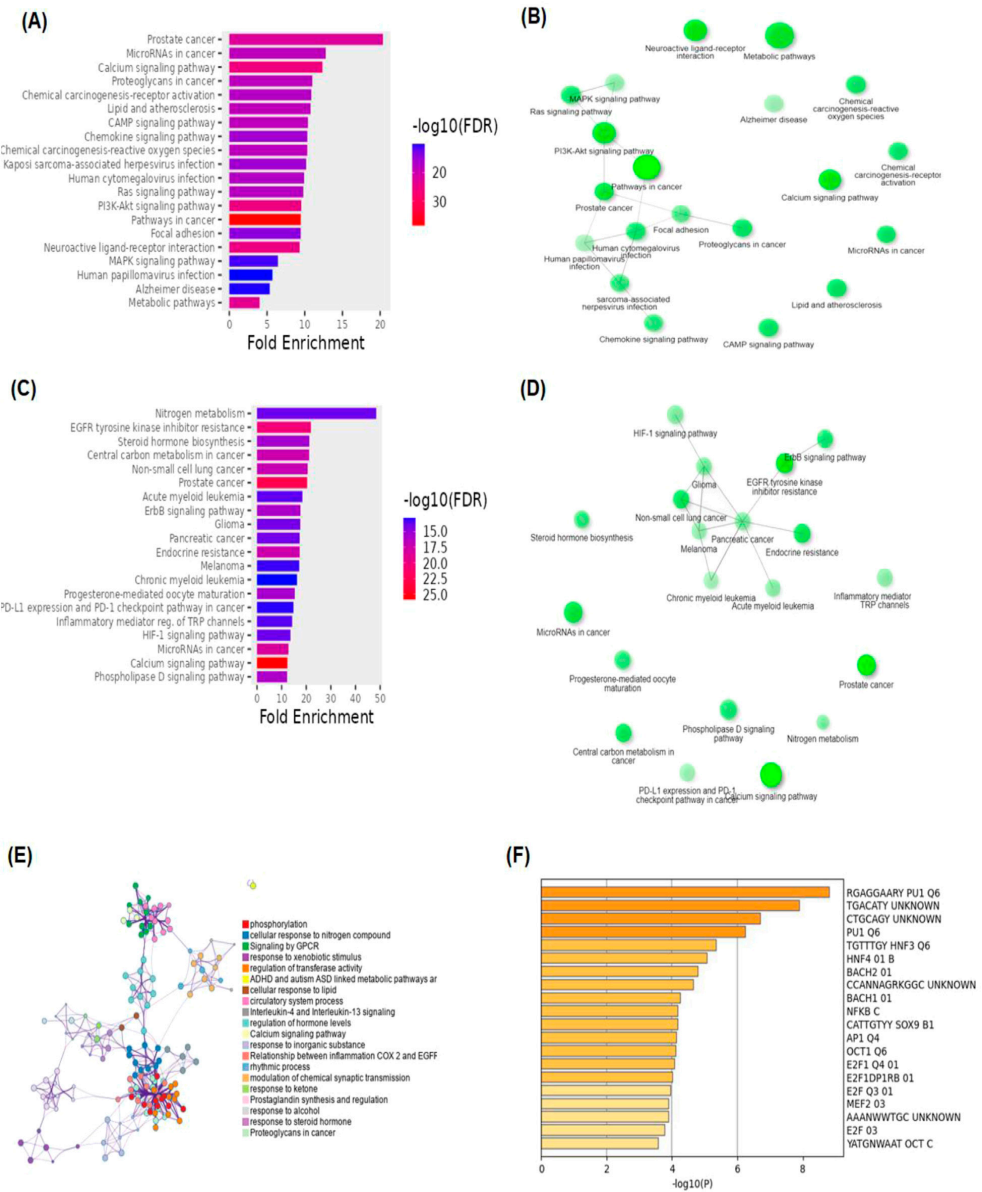
BK002 Network Pharmacology Analysis in Prostate cancer. **(A)** Gene alignment KEGG pathway of AJN and MFR, **(B)** Gene alignment KEGG pathway network, **(C)** Average equality alignment KEGG pathway of AJN and MFR, **(D)** average alignment KEGG pathway network, **(E)** overall network of AJN and MFR and **(F)** target transcription factors of AJN and MFR.

Figure 2A shows the KEGG pathway alignment for BK002 (a combination of AJN and MFR), specifically targeting DNMT1, Dicer, PD-L1, and PD-1. KEGG (Kyoto Encyclopedia of Genes and Genomes) was used to map gene interactions and pathways, highlighting which genes involved in these pathways are influenced by the bioactive compounds in AJN and MFR. The gene sets corresponding to DNMT1, Dicer, PD-L1, and PD-1 were obtained from publicly available gene databases (e.g., NCBI, KEGG). Figure 2B illustrates the interconnected network of KEGG pathways influenced by the compounds in AJN and MFR, showing how they interact with multiple prostate cancer-related pathways. Genes involved in the targeted pathways (DNMT1, Dicer, PD-L1, PD-1) were mapped with their interaction partners. The network shows direct and indirect connections between these pathways and other cancer-related genes regulated by AJN and MFR compounds. Figure 2C displays a comparative analysis of how AJN and MFR compounds affect multiple pathways on average, demonstrating the overall balance in their pharmacological action on prostate cancer pathways. KEGG pathways affected by AJN and MFR compounds were averaged based on the number of overlapping genes. This approach helps in identifying whether the two compounds target pathways with similar intensities or whether one has a stronger influence. Figure 2D highlights the average interaction network based on the aligned pathways of AJN and MFR. It aggregates the interaction data, presenting a more generalized view of how the two herbal medicines interact with the prostate cancer gene pathways. Using network pharmacology tools, the average alignment of the networks for AJN and MFR was computed. Nodes represent genes or proteins, while edges represent interactions or relationships between them.

Figure 2E presents the comprehensive interaction network of AJN and MFR compounds across all the targeted pathways in prostate cancer, including DNMT1, Dicer, PD-L1, and PD-1. It reflects the complete bioinformatics data from pathway analysis. Network pharmacology platforms (such as STITCH, STRING, or Cytoscape) were used to generate a global interaction map. This figure provides an integrative view, demonstrating the full therapeutic potential of these herbal extracts. Figure 2F focuses on the specific transcription factors regulated by AJN and MFR compounds, which are crucial in modulating the expression of DNMT1, Dicer, PD-L1, and PD-1 in prostate cancer. Transcription factors known to regulate the expression of DNMT1, Dicer, PD-L1, and PD-1 were identified using databases like TRANSFAC or JASPAR. AJN and MFR compounds were then analyzed to determine their influence on these transcription factors through docking simulations or network modeling.

### 3.2 Ethnopharmacological background of herbal medicine in prostate cancer

Traditional cancer treatments like surgical resection, radiotherapy, and chemotherapy have been fundamental in oncology. However, the advent of immunotherapy and targeted therapies has significantly enhanced cure rates. Alongside these advancements, increasing clinical and laboratory evidence supports the efficacy of herbal medicines in cancer treatment (McCubrey et al., 2017). Phytochemicals from herbal sources exhibit notable anticancer properties, complementing conventional therapies (Xiang et al., 2019). Traditional herbal medicine, with its millennia-long history, is experiencing renewed scientific interest, with modern methods validating its efficacy, safety, and mechanisms of action (Chen L et al., 2024). For instance, during the COVID-19 pandemic, around 90% of patients in China used traditional herbal medicine, achieving an 80% effectiveness rate with minimal side effects (An et al., 2021). In Asia, herbal medicine is widely used alongside conventional cancer treatments (Li et al., 2013; Xu et al., 2020). Benefits include enhanced immunity, symptom relief, and improved quality of life. Herbal extracts and formulations target multiple pathways to combat drug resistance, induce tumor apoptosis, and inhibit tumor growth (Jin et al., 2021; Lee et al., 2021; Lu et al., 2021; Wang et al., 2024).

Notably, plant-derived phytochemicals and extracts exhibit different mechanisms of effectiveness against prostate cancer (Huang et al., 2019; Livingstone et al., 2019; Wang et al., 2019; Bai et al., 2021; Ghosh et al., 2021; Singla et al., 2021; Ruksiriwanich et al., 2022; Kong et al., 2023; Peng W et al., 2023; Chen M et al., 2024; Huang et al., 2024; Ji et al., 2024). Research in systems biology will undoubtedly help modernize herbal medicine and establish the multicomponent, multitargeting approach as a new paradigm in medicine. Once identified, metabolic engineering and/or chemical synthesis can be used to produce the active constituents of a herbal medicine more efficiently (Kim et al., 2015). These treatments leverage a variety of bioactive compounds like saponins, triterpenes, phenolics, and flavonoids, known for their antimicrobial, anti-inflammatory, immunomodulatory, antioxidant, and anticancer effects (Batiha et al., 2023). The growing acceptance and integration of herbal medicines in cancer care reflect their potential to enrich and enhance therapeutic outcomes, offering a holistic approach to oncology.

### 3.3 *Achyranthes japonica* (Miq.) Nakai (AJN)

AJN, a perennial herb in the Amaranthaceae family, is prevalent in East Asia, including Korea, China and Japan (Jung et al., 2007). Traditionally used to treat edema, arthritis, mastitis, and delayed menstruation (Marcone et al., 2003). Recent studies have revealed that the phenol content of AJN reached its peak at a sowing amount of 0.5 g, while the flavonoid content is maximized at both 0.5 and 1.0 g, underscoring the ideal conditions for bioactive compound production (Kim et al., 2024). Notably, AJN is rich in phytochemicals such as saponins, inokosterone, ecdysterone, and oleanolic acid bisdemoside (Hahn and Lee, 1991; Ida et al., 1994). These compounds contribute to its diverse biological and pharmaceutical activities, including anti-inflammatory, antioxidant, antimicrobial, osteoprotective (Jung et al., 2007; Bang et al., 2012; Park et al., 2013), anti-diabetic, and anticancer (Shim et al., 2016). Dietary supplementation with AJN extract in animal models has improved growth performance, nutrient utilization, intestinal microbiota balance, and reduced excreta ammonia levels (Sun H. Y et al., 2020). Recent studies highlight the potential of AJN in various therapeutic applications. AJN, particularly in its fermented form, has shown significant effects in animal models of osteoarthritis, reducing inflammation and catabolic factors while preserving joint architecture (Kim D et al., 2020).

AJN extract demonstrated anti-allergic effects by suppressing histamine release and intracellular calcium [Ca^2+]^i elevation in FcɛRI-mediated KU812F cells in a dose-dependent manner. Flow cytometry revealed reduced FcɛRI surface expression and decreased binding of IgE to FcɛRI. Additionally, AJN extract downregulated FcɛRI α chain mRNA levels, suggesting its mechanism involves FcɛRI expression inhibition, calcium influx suppression, and histamine release reduction (Shim et al., 2016).

In a monosodium iodoacetate (MIA)-induced osteoarthritis animal model, dietary supplementation with fermented AJN (FAJN) reduced serum prostaglandin E2 (PGE2), proinflammatory cytokines, and cartilage catabolic factors such as MMP-3 and MMP-7. These findings suggest that FAJN may have therapeutic potential for managing osteoarthritis (Kim H. Y et al., 2020).

AJN root (AJNR) demonstrated specific effects on IL-6-mediated catabolic and anabolic alterations, reducing catabolic factors and recovering anabolic factors *in vitro*. In a destabilization of the medial meniscus (DMM) model, AJNR decreased cartilage erosion, subchondral plate thickness, and osteophyte size and maturity. In a CIA model, AJNR effectively inhibited cartilage degeneration, synovium inflammation, and pannus formation in the ankle and knee. Immunohistochemical analysis revealed its primary action involved suppressing IL-6-mediated matrix metalloproteinase-3 and -13 in arthritis models (Zhao et al., 2021). AJN has shown promising results in reducing NO and PGE2 production in LPS-induced cells and in decreasing MMP-3 release in TNF-α-treated cells. Fermented AJN exhibits enhanced anti-inflammatory activity and greater concentrations of active components (Lee et al., 2012). Notably, combined extracts of AJN with other herbs have shown significant anti-inflammatory effects, reducing the production of pro-inflammatory cytokines and showing potential in managing osteoarthritis and other inflammatory conditions. AJN’s inhibitory effects on NF-κB activation and ERK, JNK, and p38 phosphorylation further underscore its therapeutic potential (Bang et al., 2012).

In prostate cancer models, BK002 (AJN combined with MFR) increased DNA damage and activated p-γH2A.X, promoting ubiquitination of pro-PARP, caspase9, and caspase3, leading to apoptosis in PC3 and DU145 cells. Confocal imaging showed enhanced DNA-binding activity, while BK002 also induced CHOP activation and suppressed PI3K/AKT expression. ROS generation was critical for apoptosis, as co-treatment with NAC reduced ROS and cytotoxicity. Furthermore, BK002 significantly upregulated miR-192-5p, and its inhibition decreased apoptosis, highlighting miR-192-5p′s role in BK002-mediated anti-cancer effects. (Park et al., 2022).

Additionally, GCSB-5, another combination of AJN and other herbs, has been recommended for managing musculoskeletal conditions, including intervertebral disc disorders (Lee et al., 2017). Given the established link between inflammation and tumor progression (Ma et al., 2013), numerous clinical studies have highlighted the chemopreventive efficacy of non-steroidal anti-inflammatory drugs (NSAIDs) such as aspirin (Cuzick et al., 2009; Cuzick et al., 2015). Furthermore, the evidence supporting the potential of AJN as an anticancer agent is strong, particularly given it demonstrated anti-inflammatory properties (Table 4), which are similar to those of aspirin. Leveraging AJN in cancer therapy could offer a dual approach by targeting both inflammation and tumor growth, enhancing overall treatment effectiveness.

**TABLE 4:** Comprehensive overview of AJN’s biological effects.

Compound/Extract	Cell line/Animal model	Dose/Duration	Efficacy	Mechanism	References
Achyranthes japonica Nakai root	KU812F (human basophilic cell line)	10, 50, 100 μg/mL	Anti-Allergic effect	↓ FcɛRI, IgE, FcɛRI α chain, histamine, [Ca^2+^]*i*	Shim et al. (2016)
Achyranthes japonica Nakai	Sprague-Dawley rats (6-week-old male)	Methylsulfonylmethane (positive control) AJN 100 mg/kg body weight [b.w.], AJN 300 mg/kg b.w.)	Osteoprotective effect	↑ collagen typeI, type II ↓ PGE2, IL-1β, TNF-α, IL-6, MMP-3, MMP-7, COX-2, PGE2, aggrecan	Kim D et al. (2020)
Achyranthes japonica Nakai root	Chondrocytes 12-week-old C57BL/6J mice	*Achyranthes japonica* Nakai root (10, 20, 50 μg/mL) in presence of IL-1β (1 ng/mL), IL-6 (100 ng/mL), and TNF-α (10 ng/mL). 2 mg/kg) in 200 μL polyethylene glycol 400 (PEG-400) twice a week.10, 20, and 50 μg/mL	Anti-Inflammatory Anti-Oxidant effects	↑Aggrecan ↓ IL-1β, TNF-α IL-6. Mmp3 Mmp13, Col2a1, Sox9	Zhao et al. (2021)
Fermented AJN	RAW 264.7 SW1353, CIA-rabbits	10, 25, 50 μg/mL, and 100, 250, 500 μg/mL *in vitro*, (200 mg/kg), or JOINS (200 mg/kg) for 4 weeks in rabbit	Anti-Inflammatory, anti-Osteoarthritis effect	↓ NO, PGE_2_ TNF- α, IL-4 MMP-3	Lee et al. (2012)
Achyranthes japonica Nakai root	RAW 264.7 C57BL/6 mice	50, 100, 250, 300 μg/mL for 1 h 500 μg/mL for 8 h in the absence or presence of Act D (1 μg/mL) or CHX (1 μg/mL)	Anti-Inflammatory effect	↓ NO, iNOS, NF-κB ERK, JNK, p38	Bang et al. (2012)
BK002 (Achyranthes japonica Nakai and Melandrium firmum Rohrbach)	PC3, DU145 MDBK	PC3 with AJN (100 μg/mL) and MFR (50 μg/mL), DU145 with AJN (50 μg/mL) and MFR (25 μg/mL)	Anti-Cancer effect	↑ miR-192-5p p-γH2A.X, CHOP ↓ Bcl-2, pro-PARP, survivin, pro-caspase9, pro-caspase3, PI3K, AKT, p-AKT	Park et al. (2022)
GCSB5(Saposhnikoviadivaricata Schischek Achyranthesjaponica Nakai Acanthopanaxsessiliflorus Seem Cibotium barometz J. Smith Glycine max Merrill, and Eucommia ulmoides Oliver)	iNOS DPPH	*A. sessiliflorus* Seem, (4.55 ± 1.45 μg/mL to 7.22 ± 1.14 μg/mL) *A. japonica* Nakai, (10.52 ± 0.45 μg/mL and 12.98 ± 0.58 μg/mL) *E. ulmoides* Oliver (4.88 ± 0.27 μg/mL to 6.40 ± 0.45 μg/mL)	Anti-Oxidative Anti-Inflammatory effect	**↑**Total starch, **↓** Nitric oxide, 3-(4,5-dimethylthiazol-2-yl)-2,5diphenyltetrazolium bromide assay for cell viability	Zhao et al. (2012)

Abbreviation: ↑upregulation; **↓** downregulation; Fc epsilon receptor I, high-affinity IgE Fc receptor (FcɛRI); Immunoglobulin E (igE); Prostaglandin E2 (PGE2); Tumor necrosis factor alpha (TNF-α); Interleukin 6 (IL-6); matrix metalloproteinase-3 (MMP-3); Collagen, type II, alpha 1 (COL2A1); nitric oxide (NO): inducible nitric oxide (iNOS); Nuclear factor kappa-light-chain-enhancer of activated B cells (NF-κB); Extra-cellular Signal Regulated Kinase (ERK); c-Jun N-terminal kinases (JNKs); phosphorylated gamma histone H2A.X (γH2A.X); B-cell lymphoma 2 (Bcl-2); Poly (ADP-ribose) polymerase (PARP); Phosphoinositide 3-kinases (PI3Ks); Protein kinase B (Akt); C/EBP, Homologous Protein (CHOP); diphenyl-2-picryl-hydrazyl (DPPH).

### 3.4 *Melandrium firmum* (Siebold and Zucc.) Rohrb. (MFR)

MFR, a biennial herbaceous plant belonging to the Caryophyllaceae family, has been used in Korean folk medicine to treat a variety of ailments, including gonorrhea, anuria, and breast cancer (Evans Schultes, 1980). MFR has been found to contain a rich array of bioactive compounds, including multiple sapogenins (Chang et al., 1989), a distinctive saponin (Woo et al., 1992), flavonoids, and triterpenoids (Zheng et al., 2008). These compounds have undergone comprehensive pharmacological evaluation, revealing diverse therapeutic potentials. Sapogenins and saponins are noted for their anti-inflammatory and anticancer properties, while flavonoids exhibit potent antioxidant and vascular protective effects. Triterpenoids, on the other hand, are recognized for their immunomodulatory and anticancer activities.

Recent studies have highlighted its remarkable potential in mitigating bone loss and inhibiting osteoclast development, particularly in postmenopausal osteoporotic models. It achieves these effects by effectively suppressing RANKL-induced osteoclastogenesis, a critical driver of bone resorption. Furthermore, it downregulates pivotal signaling pathways, including NFATc1/c-Fos and TRAF6, which are essential for osteoclast differentiation and activity. This dual action not only impedes the formation of osteoclasts but also preserves bone integrity, underscoring its therapeutic promise for managing osteoporosis and related bone diseases (Kim et al., 2021). MFR demonstrated significant inhibitory effects on the expression of key genes involved in adipogenesis, including PPAR-γ, C/EBP-α, and aP2, as well as genes regulating lipogenesis, such as SREBP-1c, FAS, SCD-1, and CD36, within epididymal adipose tissue and liver tissues. These findings highlight MFR’s ability to modulate critical pathways that drive fat accumulation and lipid synthesis.

By suppressing these gene expressions, MFR effectively mitigates the molecular drivers of adipogenesis and lipogenesis, offering a promising approach to counteract high-fat diet-induced obesity. These results position MFR as a potential functional food ingredient with applications in preventing obesity and promoting metabolic health (Kim D et al., 2020). Furthermore, MFR demonstrates significant anti-inflammatory activity by targeting the 5-lipoxygenase (5-LOX) pathway, a critical enzyme in the biosynthesis of pro-inflammatory leukotrienes. Notably, several bioactive compounds isolated from MFR exhibit strong 5-LOX inhibitory effects, effectively reducing the production of leukotrienes that contribute to inflammation and related pathologies. This mechanism highlights MFR’s therapeutic potential in managing inflammatory disorders, particularly those driven by leukotriene-mediated pathways, such as asthma, arthritis, and inflammatory bowel diseases (Zheng et al., 2008).

MFR selectively induced apoptosis and cytotoxicity in human neuroblastoma cells without significantly affecting normal fibroblast cells. To our knowledge, this is the first study to demonstrate that MFR dose-dependently activates caspase signaling, mediated by the regulation of Bcl-2 family proteins. This process ultimately leads to the accumulation of fragmented DNA, highlighting its potential as a targeted therapeutic agent against neuroblastoma (Rahman et al., 2013).

**FIGURE 3:**
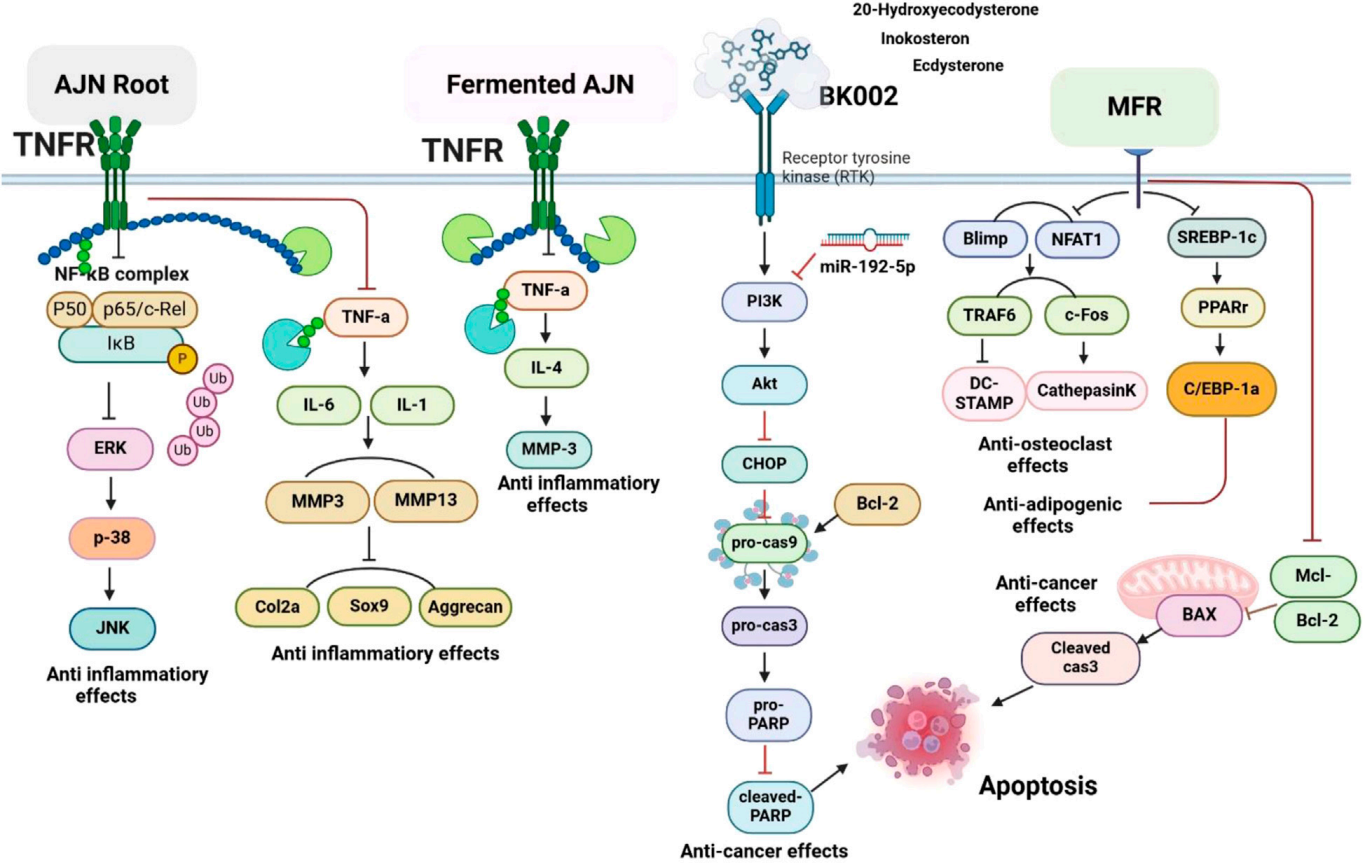
Schematic of effect with *Achyranthes japonica* Nakai (AJN) and *Melandrium firmum* Rohrbach (MFR). AJN have anti-inflammatory, antioxidant, antimicrobial, osteoprotective, anti-diabetic, and anticancer activities. MFR Inhibits osteoclast development, anti-obesity effects, potent anti-inflammatory properties and anti-cancer effects. BK002 (combination of AJN and MFR), showing synergistic effects. Enhanced anticancer effects, including inhibition of NF-κB activation and ERK, JNK, and p38 phosphorylation, contributes to the diverse pharmacological effects.

MFR exhibits a wide range of beneficial effects across various experimental models, demonstrating anti-osteoclast, anti-adipogenic, anti-cancer, and anti-inflammatory properties, as shown Table 5. The subsequent sections will introduce specific flavonoids, exploring their unique roles and mechanisms as transformative agents in cancer treatment. Figure 3 illustrates the key phytochemicals and extracts derived from AJN and MFR, highlighting their structural diversity and therapeutic potential.

**TABLE 5:** Diverse biological effect of MFR.

Compound/Extract	Cell line/Animal model	Dose/Duration	Efficacy	Mechanism	References
*Melandrium firmum* Rohrbach	RAW 264.7 MC3T3-E1, ovariectomized (OVX) rat	RANKL (100 ng/mL), MFR (12.5, 25, 50, 100 μg/mL) for 5D Inhibition- RANKL (100 ng/mL), MFR (50,100 μg/mL), vitexin (0.0753, 0.147 μg/mL)	Anti-osteoclast effects	↓ CA2/*Ca2*, TRAF6 NFATc1/c-Fos Acp5, Atp6v0d2 DCSTAMP/Dc stamp*,* Oscar, c-Src, Blimp-1/Prdm1	Kim et al., (2021)
*Melandrium firmum* Rohrbach	3T3-L1. C57BL/6N	10 μg/mL insulin, 0.5 mM 3-isobutyl-1-methylxanthine, 1 μM examethasone, 10 and 50 μg/mL	Anti-adipogenic Anti-lipogenic effects	↓ PPAR-γ, C/EBP-α, aP2, SREBP-1c, FAS, SCD-1, CD36	Kim H. Y et al. (2020)
*Melandrium firmum* Root extract	SH-SY5Y, B103, NIH3T3	20, 25, and 30 μg/mL	Anti-cancer effects	↓Mcl-1, Bcl-2 ↑Cleaved caspase-3, Bax	Rahman et al. (2013)
*Melandrium firmum* Rohrbach	Bone marrow-derived mast cells (BMMCs)	IC_50_ 21.04 μM 42.30 μM 32.82 μM,17.18 μM	Anti-inflammatory effects	↓COX-2, 5-LOX	Zheng et al. (2008)

Abbreviation: ↑upregulation; **↓**downregulation; Carbonic anhydrase 2 (CA2/*Ca2*; TNF, receptor-associated factors (TRAFs); Nuclear Factor Of Activated T Cells 1 (*NFATC1*); tartrate-resistant acid phosphatase 5 (*ACP5*/TRAP); ATPase, H+ transporting, lysosomal 38kDa, V0 subunit d2 (*Atp6v0d2)*; *dendritic cell specific transmembrane protein* (DC STAMP); dendritic cell specific transmembrane protein (*DC STAMP*); *Osteoclast*-associated Ig-like receptor (Osca); B-lymphocyte-induced maturation protein 1 (BLIMP1); PR, domain zinc finger protein 1(Prdm1); Peroxisome Proliferator-Activated Receptor (PPAR); CCAAT/enhancer binding protein (C/EBP); Sterol regulatory element-binding transcription factor 1 (SREBF1); fatty acid synthase (FAS); Scd1 stearoyl-Coenzyme A desaturase 1 (SCID-1); Cyclooxygenase-2 (COX-2); *5*-*lipoxygenase* (5-LO).

### 3.5 Phytochemistry: biochemical scaffolds with multifaceted therapeutic potential

Several categories of bioactive compounds have been identified, including flavonoids, terpenes, alkaloids, phenolic acids, and steroids, each contributing to a diverse range of pharmacological activities (Wei et al., 2023).

It has been identified ecdysone and cyasterone as the primary steroids in the methanolic extract of the whole plant. Further investigations were conducted Sabri et al. (1981) demonstrated the presence of three major steroids cyasterone, ecdysterone (C27), and ajugasterone C across the roots, stems, and leaves, underscoring the plant’s significant pharmacological potential (Bouyahya et al., 2020). Detailed information on steroids, particularly ecdysterone (C27), will be elaborated in the subsequent section, 3.6 Phytochemical Composition of AJN and MFR, providing deeper insights into their significance and potential applications.

Flavonoids, a diverse group of phytonutrients found in many fruits and vegetables, are well-recognized for their therapeutic properties, including anti-inflammatory, antioxidant, and anticancer activities. These compounds have a biochemical structure characterized by a benzo-γ-pyrone framework that allows them to scavenge free radicals (Heijnen C et al., 2001), chelate metal ions, and modulate key cellular enzymes involved in growth and apoptosis. The structural characteristics of flavonoids, particularly the catechol moiety present in many highly active flavonoids such as luteolin, morin, apigenin, chrysin, and galangin, play a crucial role in their biological activity.

The catechol structure, typical on the aromatic B-ring of flavonoids, comprises two adjacent hydroxyl groups and provides substantial antioxidative potential (Tse et al., 1976; Haenen et al., 1991). This structure enables the formation of intramolecular hydrogen bonds and stabilizes the radical form of the flavonoid, facilitating the donation of a hydrogen atom, a critical step in free radical scavenging (Heijnen C. G et al., 2001; van Acker et al., 2001). The effectiveness of the catechol structure is further evidenced by its impact on the π-conjugation system of flavonoids. These compounds are characterized by their unique benzo-γ-pyrone structure, which facilitates a range of biological activities, including their ability to act as effective scavengers of free radicals (Marković et al., 2017; Masek et al., 2017; Amić et al., 2018). The conjugation extends from the B-ring across the C2-C3 double bond into the carbonyl group on the C-ring, enhancing the overall stability and radical-scavenging capacity of these molecules compared to their saturated counterparts, such as flavanones (Zheng et al., 2019; Bors et al., 1990; Van Acker et al., 1996; Heijnen C et al., 2001; Spiegel et al., 2020).

Integrating the principles of systems biology and multitargeting therapies, including those derived from herbal medicines, holds promise for enhancing treatment efficacy and addressing therapeutic resistance through synergistic interactions at multiple biological levels (Li et al., 2011). This approach could modernize herbal medicine and establish a new paradigm in medicine, enabling more systematic investigations and large-scale production of pure active compounds for various therapeutic applications (Kim et al., 2015). Saponins, triterpenes, phenolic compounds, isoflavones, and flavonoids are notable for their diverse bioactivities, particularly in cancer therapy.

Saponins, found in many plants, exhibit immunostimulatory, anti-inflammatory, and anticancer properties by inducing apoptosis and inhibiting tumor proliferation through signaling pathway modulation (Mieres-Castro and Mora-Poblete, 2023). Certain triterpenes, characterized by their 30-carbon structure, can suppress cancer growth, induce apoptosis, and inhibit angiogenesis (Xu et al., 2018). With a novel mechanism involving the interaction of the triterpene glycoside’s carbonyl oxygen with the Fe/S center of the mitochondrial respiratory chain, hydrogen peroxide is produced, which leads to the opening of the mitochondrial transition pore (Salvi et al., 2003; Fiore et al., 2004). This action highlights their unique potential in cancer therapy.

Phenolic compounds, identified by hydroxyl groups attached to an aromatic ring, offer strong antioxidant properties, protecting cells from oxidative stress, a critical factor in cancer development. They also demonstrate anti-inflammatory and anticancer activities by modulating signaling pathways and inducing apoptosis in cancer cells (Sadhu et al., 2022).

Isoflavones, a type of flavonoid primarily found in legumes, act as phytoestrogens with estrogen-like effects. They have a unique 3-phenylchromen-4-one structure, which enables them to modulate estrogen receptors and inhibit key enzymes such as tyrosine kinase and topoisomerase-II, providing significant therapeutic benefits (Russo et al., 2016; Mukund et al., 2017).

Flavonoids, with a distinct benzo-γ-pyrone structure, exhibit therapeutic properties including anti-inflammatory, antioxidant, and anticancer effects. Their ability to scavenge free radicals, chelate metal ions, and modulate essential cellular enzymes involved in growth and apoptosis underlines their efficacy. These compounds interact with multiple molecular targets, making them versatile agents in cancer therapy (Kumar et al., 2023).

Incorporating herbal medicine not only capitalizes on its vast arsenal of bioactive compounds but also enhances the precision of cancer treatments by harnessing natural synergies. This approach proactively counters the emergence of drug resistance, shifting from a reactive to a preventative treatment model. Ultimately, integrating the traditional wisdom of herbal medicine with advanced systems biology and innovative drug development is poised to revolutionize our approach to cancer therapy, leading to more effective, sustainable, and holistic treatment outcomes. As we delve into their specific roles and mechanisms, we will explore how these candidates could disrupt cancer progression, opening new avenues in the fight against this complex disease.

Table 6 highlights the potential binding interactions and energies between various bioactive compounds and key proteins involved in cancer, offering insights into their therapeutic potential.

**TABLE 6:** DNMT1, PD-L1, Dicer, PD-1, and Apoptosis Inducing Factor Binding amino acid as well as binding energy.

Compound and structure	Component	Target protein	Binding amino acids	Binding energy (kcal/mol)
Luteolin 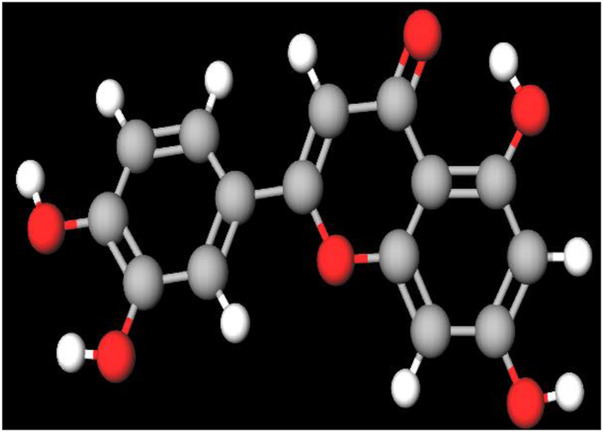	Catechol moiety	DNMT1	Asp119, Ser120, Tyr121	−8.5
Morin 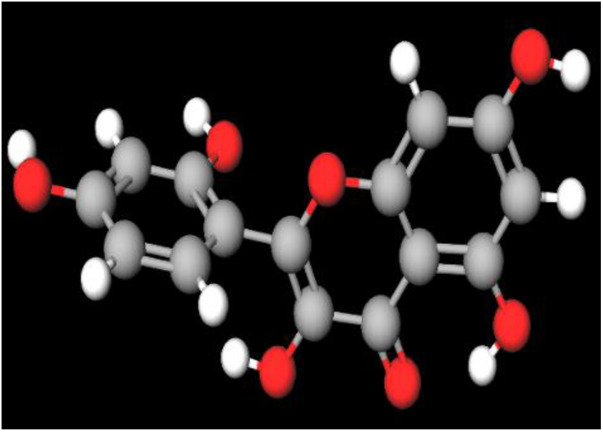	Catechol moiety	PD-L1	Leu15, Arg17, Phe19	−7.8
Apigenin 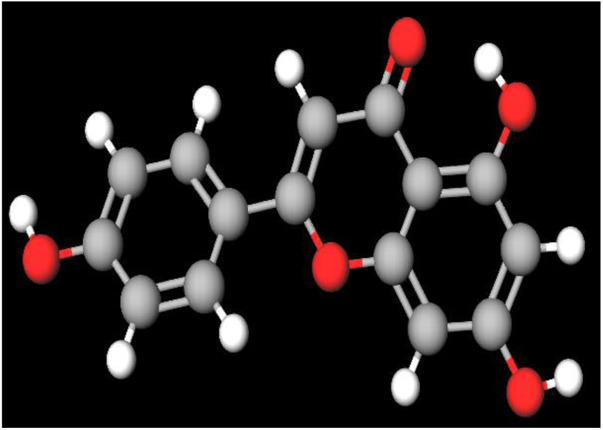	C2–C3 double bond	Dicer	Phe62, Thr64, Ser66	−8.2
Chrysin 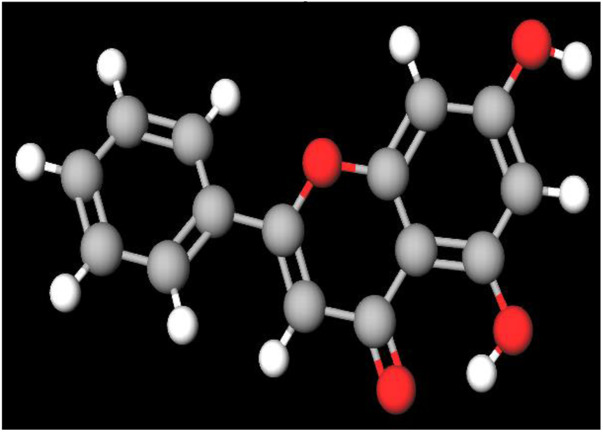	Carbonyl group on the C-ring	PD-1	Glu12, Asp18, Ser22	−7.9
Galangin 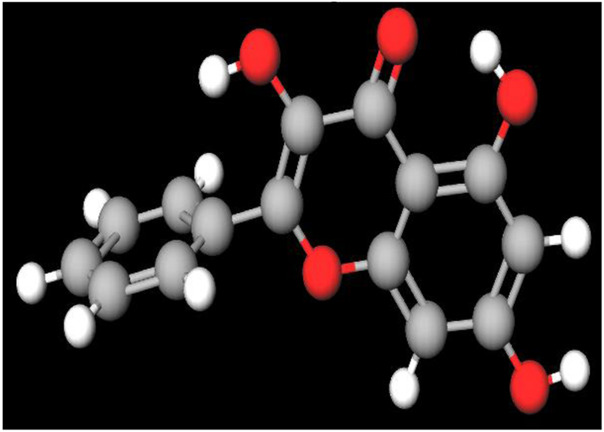	Benzo-γ-pyrone	Apoptosis Inducing Factor	Gly126, Val128, Arg130	−8.4
Saponins 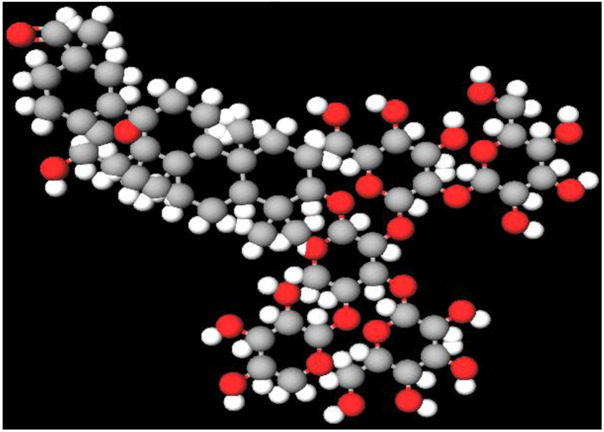	Glycoside backbone	PD-1	Lys22, Gly26, Arg28	−8.1
Triterpenes 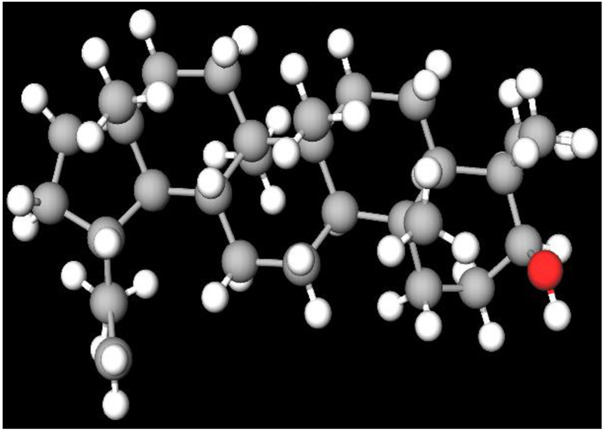	Carbonyl oxygen	Apoptosis Inducing Factor	Ala150, Lys154, Ser158	−8.7
Phenolic Compounds 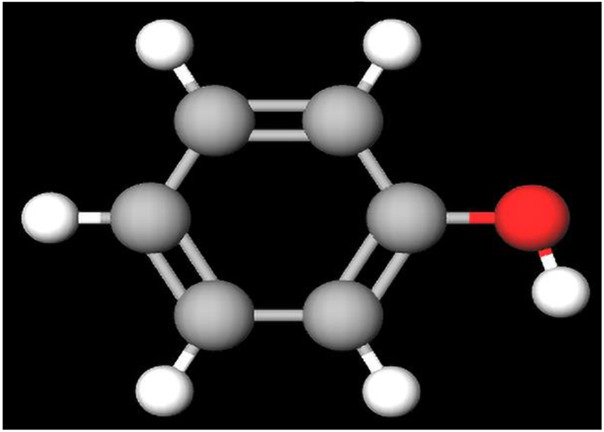	Hydroxyl groups	DNMT1	Thr140, Asp143, Glu145	−8.9
Isoflavones 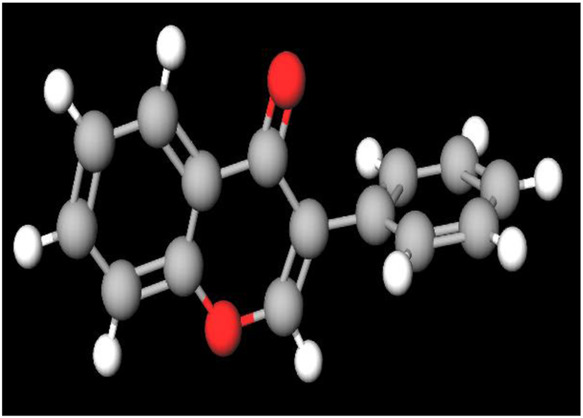	3-Phenylchromen-4-one	PD-L1	Met23, Val25, Ser27	−7.6
Flavonoids 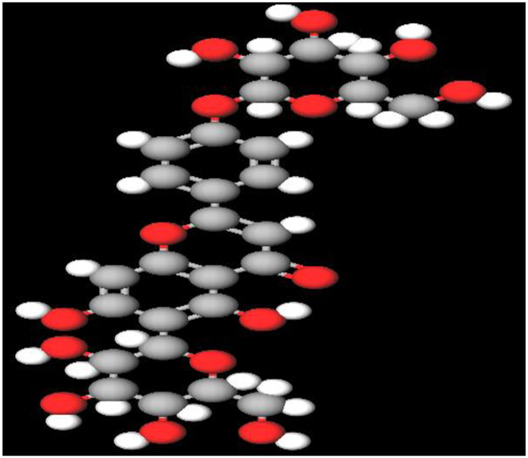	Benzo-γ-pyrone	Dicer	His34, Asp36, Lys40	−8.3

### 3.6 Key steroidal components of AJN and MFR

Phytosterols have shown promising potential in combating various cancers, including breast, prostate, lung, liver, stomach, and ovarian cancers. Research has demonstrated their ability to inhibit the growth and proliferation of cancer cells, particularly in liver, prostate, and breast cancers, highlighting their therapeutic potential in cancer prevention and treatment (Ramprasath and Awad, 2015). AJN and MFR are two traditional herbs with promising pharmacological properties. Recent research has illuminated the phytochemical compositions of these plants, underscoring their potential in enhancing health and treating diseases, including cancer.

AJN is renowned for its diverse bioactive compounds, which contribute to its many therapeutic applications. One standout compound is ecdysterone, a naturally occurring steroid hormone that has garnered attention for its ability to bind to estrogen receptors, a mechanism that may underpin its anabolic effects. In animal studies, ecdysterone has outperformed even banned anabolic agents like metandienone, although human studies remain limited (Isenmann et al., 2019).

Steroids, characterized by their four-ring core structure, are ubiquitous in the biological world, serving crucial roles from maintaining cell membrane integrity to signaling. Ecdysteroids are synthesized by about 6% of plant species as a defense mechanism against insect herbivores. These compounds have a distinctive biochemical configuration that grants them significant biological activity, including the regulation of gene expression and various metabolic effects (Shuvalov et al., 2020). Among these substances, 20-hydroxyecdysone and ecdysteroids are the most prevalent, and control arthropod reproduction, development, diapause, and molting (Bortolozzi et al., 2020). Ecdysteroids are polyhydroxylated steroids with a four-ring core structure like cholesterol and steroid hormones. Ecdysterone has a distinctive 14α-hydroxy group and a seven-ene function (Miyata et al., 2007). Despite their promise, the exploration of ecdysteroids in cancer therapy is still in its infancy and warrants further research (Dinan, 2001; Lafont and Dinan, 2003; Mostrom and Evans, 2011; Martins et al., 2013; Ling et al., 2019; Ju et al., 2021).

MFR also exhibits a rich phytochemical profile with significant therapeutic potential. The aerial parts of MFR have yielded novel anthraquinone dimers, such as melrubiellins A−D that have demonstrated substantial cytotoxic effects in specific cancer cells, suggesting MFR’s potential as an anticancer agent (Zhang et al., 2015a; Zhang et al., 2015b). Another standout compound isolated from MFR is α-spinasterol, which has shown efficacy in preventing benign prostatic hyperplasia (BPH) in animal models. This compound, also isolated from the aerial parts of *Doellingeria scabra* (Thunb.) Nees (Asteraceae), exhibits several pharmacological actions, including anticancer and anti-inflammatory effects. Furthermore, the anti-inflammatory properties of MFR are supported by the presence of potent flavonoids like schaftoside, homoorientin, cytisoside, vitexin, and isovitexin. These compounds were identified and quantified using advanced HPLC-PDA techniques, highlighting MFR’s robust anti-inflammatory and antioxidant potential (Lee et al., 2014).

Both AJN and MFR stand out due to their rich phytochemical compositions and diverse therapeutic properties. From ecdysteroids in AJN that exhibit anabolic and anticancer activities to the novel anthraquinones and flavonoids in MFR that show significant cytotoxic and anti-inflammatory effects, these herbs hold immense promise. Their bioactive compounds offer a natural, multifaceted approach to enhancing health, potentially transforming modern therapeutic practices, especially in oncology. Further research into these plants’ molecular mechanisms will undoubtedly pave the way for their integration into contemporary medicine.

The moderate toxicity profiles of 20-hydroxyecdysone (20-HE) and inokosterone, combined with the absence of severe adverse effects, underscore their potential as safe therapeutic agents. Their application in medicine, particularly in contexts requiring reduced toxicity, presents a promising avenue for future research and drug development. In contrast, traditional chemotherapeutic agents, while effective, carry significant risks that necessitate careful consideration and management in clinical settings. Clinical experiments involving AJN and MFR have not yet concluded, but it is anticipated that clinical trial results will reflect clinical efficacy.

Ecdysteroids have been applied in rat models for the osteogenic differentiation of mesenchymal stem cells (BMSCs) and ovariectomy (OVX)-induced osteoporosis (OP). Also examined was the potential promotion of IS on the osteogenic differentiation of BMSCs *in vitro* by assessing the cell viability, mineralization capacity, and collagen I, ALP, and OCN expression levels. Furthermore, IS treatment led to an upregulation of the BMP2/smad1/RUNX2 pathway expression in BMSCs (Chen Y et al., 2023).

Ecdysteroid-containing preparation Serpisten and inokosterone have been investigated, both prior to and following chronic low intensity gamma-irradiation in a mouse model. Results indicate the dose-dependent antiradiation characteristics of these materials. Serpisten prevented the reduction gain in body mass caused by radiation. Following radiation exposure, treatment with this preparation at a dose of 50 mg/kg resulted in normalization of the phospholipid composition of the mouse liver and blood erythrocytes for most of the parameters under investigation. Furthermore, the ability of Serpisten to break down peroxides was demonstrated *in vitro*. Due its specific anabolic characteristics, inokosterone also led to the normalization of liver phospholipid composition (Shevchenko et al., 2007).

Four insect pests (*L. cuprina*, *Myzus persicae*, *Bemisia tabaci*, and *H. armigera*) have their ligand binding domains (LBDs) from the EcR and USP proteins isolated as recombinant heterodimers. The hinge sections of LBD heterodimers, or DE/F heterodimers, were included in a binding that ranged from 0.7 to 2.5 nM. The K(i) values for the ligands of ecdysteroid and dibenzoylhydrazine varied from 0.1 nM to >448 μM. A recombinant *Helicoverpa armigera* LBD heterodimer lacking D-regions (an E/F heterodimer) had K(d) and K(i) values that were around four times greater than those of its DE/F counterpart. Rate constants for the LBD heterodimer of *Lucilia cuprina* were estimated. (Graham et al., 2007).

Table 7 provides insights into the therapeutic potential of important proteins implicated in BK002 activity, as well as the possible effects of ecdysterone, 20-HE, and inokosterone.

**TABLE 7:** Pharmacological activities of ecdysterone, 20-HE, and inokosterone.

Compound /Extract	Cell line/Animal model	Dose/Duration	Efficacy	Mechanism	References
Ecdysterone	MCF7, MDA-MB-231, MDA-MB-468, DF2, WI-38, Osteoporotic rats	50 μM	Anti-Cancer effect	↓LC3B, p62, basic OCR stressed OCR stressed ECAR	Shuvalov et al. (2020)
20-HE	A549, H1299, H460	(0.1–100 µM) for 1.5 h 10 µM of 20E for 24 h, 48 h	Anti-Cancer effect	*↑*Gpx3*, *Gpx4*, *Gpx6*,* Gsr*,* Gss*,* Prdx1*, *Prdx5*, *Prdx6*,* Sod1*, *Sod2*, *Sod3, G0/G1 arrest ↓ROS, Notch3, HSF1, mTOR, SOX12, KLF16, ABCB6, ABCC1, TGF-β, MAPK, HK2, LDHA, SHMT2, MTHFD2, c-Myc, ATF4. ALDH, CD44, Oct4, c-Kit, Nestin	Shuvalov et al. (2023)
20-HE	MCF7, T-47D, MDA-MB-231	200 μM	Proapoptotic, Pro Autophagic effect	*↑* mTOR, Bax, LC3, p62, G2/M ↓Bcl-2, PARP, caspase-3	Romaniuk-Drapała et al. (2021)
20-HE	J82, 5637, T24, SW780, UMUC3 SV-HUC-1 Xenograft mouse	2.5, 5, 10 μM	Anti-Cancer effect	↓caspase-3, USP21 NF-κB/p65, N-cadherin, IKBKB, PARP1, RAB21, FBXL14, RNF168 ZEB-1, Vimentin, MMP13	Ma et al. (2024)
20-HE	A-549, SW-620	1.79 µM, 1.83,1.85 µM	Anti-Cancer effect	*↑*ROS, Bax ↓BCL-2, Caspase-3, MMP	Lone et al. (2023)
20-HE	HeLa-IL-6	3.5–6.2 μg/mL	Anti-Inflammatory effects	↓NF-κB, TNF*α*	Peschel et al. (2011)
Inokosterone	BMSCs Female SD rats (250 ± 5 g body weight)	50, 100 200 mg/L, 2, 4 mg/kg	Osteogenic effect	*↑*BMP2, Smad1, RUNX2, collagen I, ALP, OCN.	Chen Y et al. (2023)
Inokosterone	22.6 cGy chronic gamma-irradiation of mice	50 mg/kg	Anabolic effect	*↑*peroxidase (brain, liver), LPO	Shevchenko et al. (2007)

Abbreviation: ↑upregulation; **↓**downregulation; Glutathione peroxidase (GPX); Glutathione reductase (GSR); Glutathione synthetase (GSS); Superoxide dismutase (SOD); Reactive oxygen species (ROS); Heat shock factor 1 (Hsf1); mammalian target of rapamycin (mTOR); SRY-box, transcription factors (SOX); Kruppel like factor 16 (KLF16); ATP-binding cassette super-family B member 6 (ABCB6); ATP, binding cassette subfamily C member 1 (ABCC1); Transforming growth factor-beta, (TGF-β); Mitogen Activated Protein Kinase (MAPK); Hexokinase 2 (HK2); Lactate dehydrogenase A (LDHA); Serine hydroxy methyltransferase 2 (SHMT2); Methylenetetrahydrofolate dehydrogenase/cyclo hydrolase (MTHFD2); Activating Transcription Factor 4 (ATF4); Aldehyde Dehydrogenase 2 (ALDH2); BCL2 Associated X, Apoptosis Regulator (BAX); Nuclear factor kappa-light-chain-enhancer of activated B cells (NF-κB); runt-related transcription factor 2 (RUNX2); Bone Morphogenetic Protein 2 (BMP-2); Alkaline phosphatase, (ALP); Osteocalcin (OCN); specific protease 21 (USP21), matrix metalloproteinase-13 (MMP13).

### 3.7 Molecular docking and ΔG values of ligands against proteins

To understand the interactions between ecdysterone, inokosterone, 20-HE, and prostate cancer-related enzymes such as 5α-reductase and CYP17, molecular docking studies were carried out. These studies calculate the binding affinities (ΔG values), which indicate how strongly a compound can bind to a specific target protein, as shown in Table 8. Lower ΔG values represent stronger binding, suggesting higher inhibitory or agonistic potential. These ΔG values suggest that while ecdysterone, inokosterone, and 20-HE are not as potent as classical inhibitors of 5α-reductase or CYP17, they still exhibit moderate affinity, individually. As shown in Table 8, the synergistic effect of BK002, which contains all these compounds, may contribute to the reduction of the androgen effect, which is beneficial for prostate cancer treatment by reducing the availability of potent androgens like DHT.

**TABLE 8:** Structural and functional similarity between ecdysteroids and human steroid hormones.

Compound and structure	Binding mode	Target enzyme	Binding amino acids	ΔG value (kcal/mol)
Ecdysterone 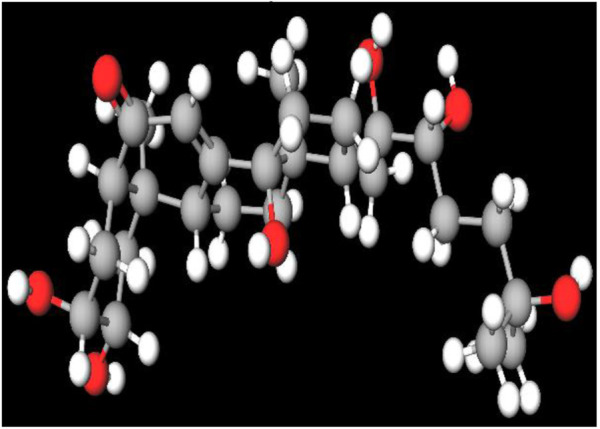	Steroid Core Binding	5α-Reductase	Tyr91, Met106, Ala220	−7.2
Inokosterone 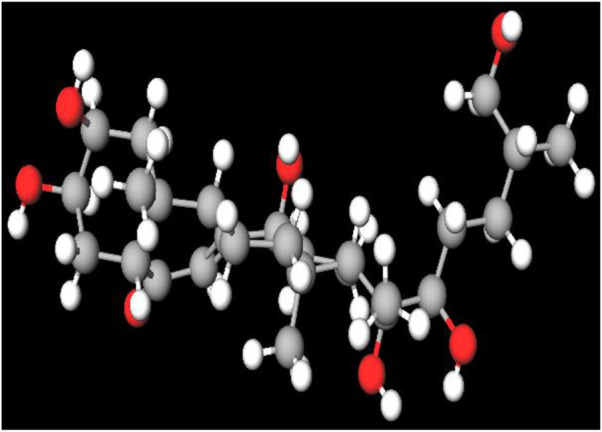	Steroid Core Binding	5α-Reductase	Arg88, His231, Leu253	−6.8
20-Hydroxyecdysone (20-HE) 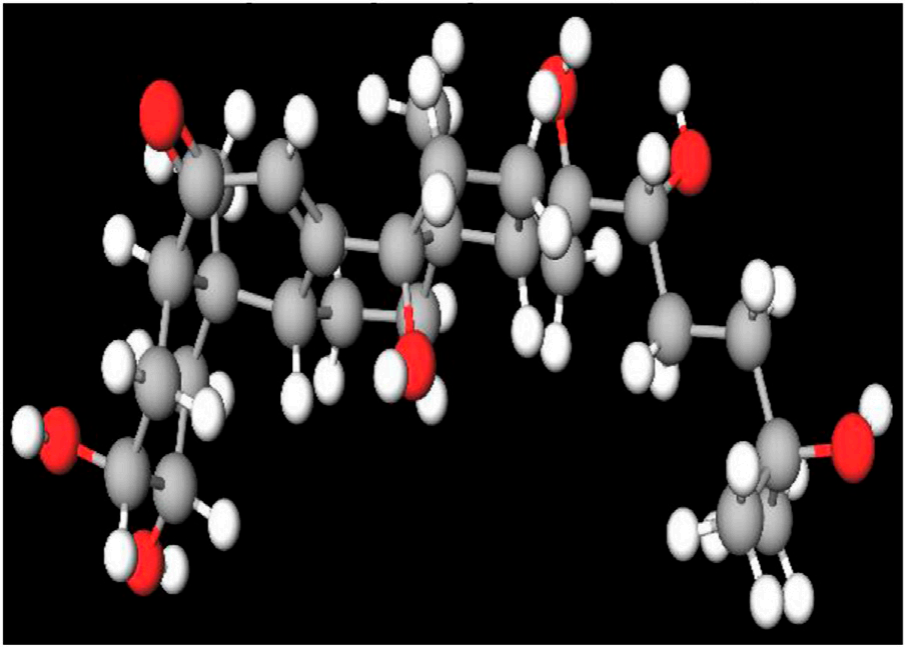	Hydroxyl Group Interaction	5α-Reductase	Ser102, Val169, Glu215	−7.4
Ecdysterone 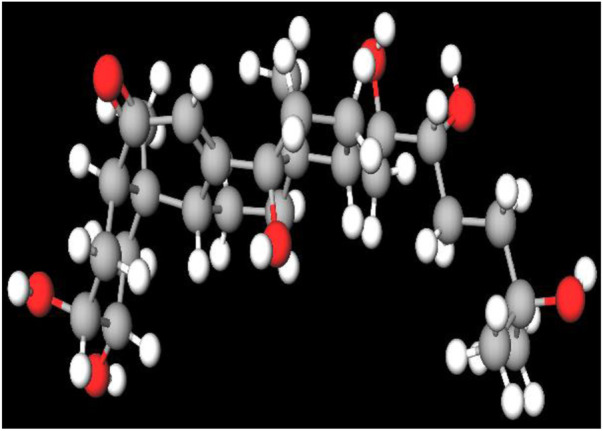	Hydroxyl Group Interaction	CYP17	Phe114, Thr306, Asn202	−7.1
Inokosterone 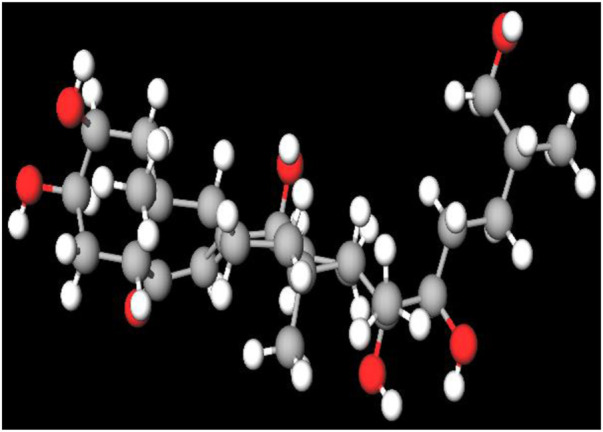	Steroid Core Binding	CYP17	Asp298, Tyr60, Val136	−6.7
20-Hydroxyecdysone (20-HE) 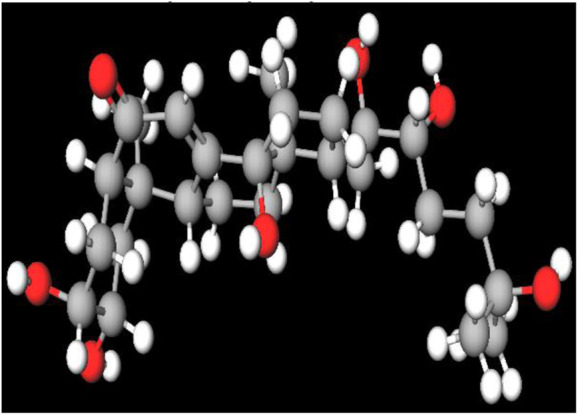	Hydroxyl Group Interaction	CYP17	Gly216, Thr210, Lys91	−7.3

### 3.8 Docking study overview

This study focuses on three critical proteins, DNMT1, Dicer, and PD-1, which play pivotal roles in various signaling pathways and biological processes, making them significant targets for cancer therapeutics. DNMT1 is crucial for maintaining DNA methylation patterns during cell division. It plays a significant role in cancer development by suppressing tumor suppressor genes through epigenetic modifications (Liu et al., 2024). Understanding these modifications can provide new avenues for cancer treatment beyond known genetic mutations (Park, 2023). Dicer, an endonuclease from the RNase III family, converts precursor microRNAs into mature miRNAs (Zhang et al., 2004). Low levels of miRNAs in various cancers are often linked to poor expression or malfunction of Dicer, implicating it in cancer progression (Kumar et al., 2009; Martello et al., 2010; Faggad et al., 2012; Khoshnaw et al., 2012). Additionally, Dicer is involved in the nucleus in processes such as chromatin remodeling, epigenetic modification, and DNA damage repair (Castel and Martienssen, 2013; Doyle et al., 2013; White et al., 2014; Burger and Gullerova, 2015; Bronisz et al., 2020). PD-1 and its ligand PD-L1 are integral to the cancer-immunity cycle. The development of anti-PD-1/PD-L1 antibodies has shown therapeutic success, emphasizing the importance of these interactions in cancer treatment (Zhao et al., 2023).

This study aims to elucidate the molecular mechanisms underlying the therapeutic effects of AJN and MFR on cancer. By identifying differential expression genes associated with cancer and validating the active ingredients and their targets through bioinformatics and molecular docking analyses, this research seeks to uncover the potential of AJN and MFR as effective herbal treatments for cancer. Additionally, *in vitro* experiments will further validate these findings, emphasizing the multi-component, multi-target, and multi-pathway strategies employed by these herbal medicines. The docking study was conducted using the CB-Dock2 method, an enhancement of the original CB-Dock server, for the blind docking process guided by binding cavity detection. This method integrates cavity identification, docking, and alignment with homologous templates. CB-Dock2 automates the identification of potential binding sites on proteins, determining their central points and dimensions, adjusting the docking box dimensions to fit specific ligands, and executing molecular docking via AutoDock Vina. The process includes.

### 3.9 Amino acid residue interaction on selected ligands against DNMT1, dicer, and PD-1 with binding affinities and entropy data

Table 9 presents the binding affinities and entropy contributions of various ligands when docked against the four key proteins DNMT1, Dicer, PD-1, and PD-L1. The binding affinity values (ΔG) indicate the strength of the interaction between the ligand and the protein, with more negative values suggesting stronger binding. The binding affinity (kcal/mol) values reflect the predicted strength of interaction between the ligand and the target protein. Lower (more negative) values indicate stronger binding affinity. Entropy (kcal/mol) contribution reflects the changes in the system’s disorder upon ligand binding. The values typically range from negative, indicating a stabilizing contribution of binding. Protein residues represent the amino acids in each target protein that are interacting with the ligands. The specific residues involved in the interaction are listed for each protein.

**TABLE 9:** Amino acid residue interaction on selected ligands against DNMT1, Dicer, PD-1, and PD-L.

Ligand	DNMT1 residues	Binding energy (kcal/mol)	Dicer residues	Binding energy (kcal/mol)	PD-1 Residues	Binding energy (kcal/mol)	PD-L1 Residues	Binding energy (kcal/mol)
Ecdysterone	Ala180, Lys182, Tyr185	−10.5	Phe91, Gly93 Ser95	−9.8	Ile26, Lys28, Val30	−9.4	Thr31, Asn33, Pro35	−9.1
Inokosterone	Ser170, Asp172, Ala174	−10.0	Thr83, Glu85 Asp87	−9.6	Ser34, Gly36, Thr38	−9.2	Val39, Ser41, Leu43	−9.0
20-Hydroxy ecdysone (20-HE)	Ala180, Lys182, Tyr185	−10.2	Phe91, Gly93 Ser95	−9.7	Ile26, Lys28, Val30	−9.5	Thr31, Asn33, Pro35	−9.2
Finasteride	Ser155, Thr157, Gly159	−9.4	Ala85 Thr87 Glu89	−9.3	Val63, Thr65, Asn67	−8.5	Ser42, Gly44, Phe46	−8.4
Abiraterone Acetate	Leu130, Ile135, Phe138	−8.9	Val42 Pro44 Ala46	−9.0	Gly12, Lys14, Val18	−8.2	Tyr29, Arg31, Met33	−8.1
Enzalutamide	Gly120, Ser122, Phe124	−9.8	Thr72, Lys74 Asp76	−9.4	Glu22, Asp24, Ser26	−8.9	Leu32, Arg34, Phe36	−8.7
Apalutamide	Leu130, Ile132, Val134	−9.6	Val52 Pro54 Gly56	−9.2	Phe62, Thr64, Asn66	−8.6	Met25, Val27, Ser29	−8.3
Darolutamide	Thr145, Ala147, Lys149	−9.7	Asp65, Ala67 Gly69	−9.5	Ser33, Gly35, Lys37	−8.8	Glu21, Asp23, Lys25	−8.5

This table compares various known prostate cancer therapeutics, such as finasteride, abiraterone acetate, enzalutamide, apalutamide, and darolutamide, alongside natural compounds like ecdysterone, inokosterone, and 20-hydroxyecdysone (20-HE), with binding information against DNMT1, Dicer, PD-1, and PD-L1.

### 3.10 Visualization of amino acid residue interaction with ecdysterone and inokosterone

Table 10 details the amino acid residue interactions for selected ligands against DNMT1, Dicer, PD-1, and PD-L1, along with their corresponding binding affinities, a powerful tool to understand how these compounds interact with their target proteins. Helping to elucidate the precise molecular interactions, the data provides insights into the potential efficacy and mechanisms of action for these compounds in therapeutic contexts, particularly in cancer therapy.

**TABLE 10:** Selected ligands against DNMT1, Dicer, PD-1, and PD-L1with binding affinities.

Protein/Ligand	Docking score	Amino acid interaction	Visualization	
			Docking	Binding site
**DNMT-1/**Ecdysterone	−10.5	**Chain B**: ILE1042 ASN1043 TYR1078 SER1079 GLN1080 GLY1081 GLY1082 PRO1083 ASP1084 ARG1085 PHE1086 GLU1156 GLN1160 TYR1307 GLY1308 VAL1309 LEU1329 PHE1330 PRO1331 GLU1332 LEU1334 HIS1335 ASN1356 ARG1359 PHE1365 TRP1398 PHE1399 GLN1402 LEU1403 ARG1404 GLY1405 GLY1560 PHE1561 PRO1562 PRO1584 PRO1585 LYS1588 ALA1589 ILE1590 LEU1592 GLU1593 LYS1595 LEU1596	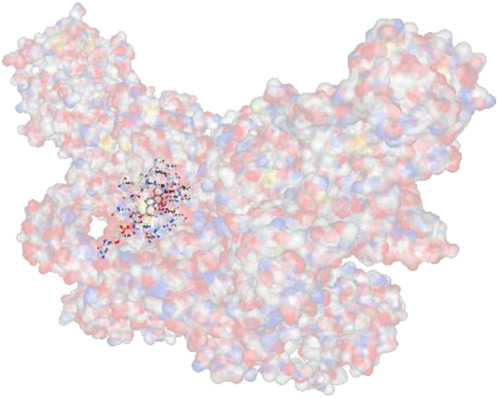	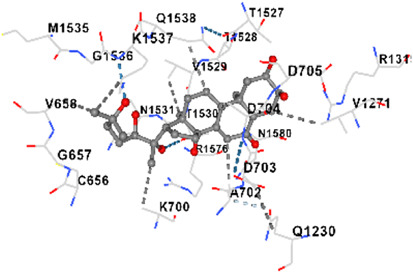
**Dicer/**Ecdysterone	−9.8	**Chain C**: GLU336 LEU337 ASP340 GLU369 ARG372 GLU374 ALA375 TYR378 SER399 ASP404 GLU407 GLU435 HIS436 SER437 LYS438 SER478 PHE480 CYS481 SER482 SER483 ARG484 SER486 **Chain D**: GLN333 GLU336 LEU337 ASP340 GLU369 ARG372 GLU374 ALA375 TYR378 SER399 ASP404 GLU407 LYS657 GLU673 THR677 ARG678 ARG680 GLN681	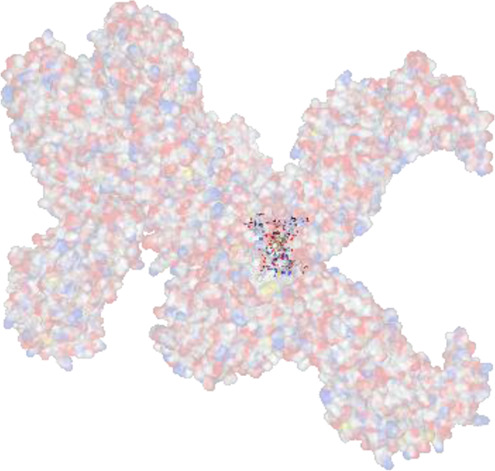	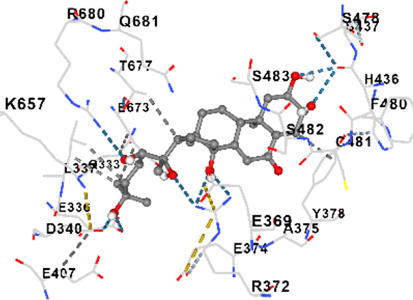
**PD-1/**Ecdysterone	−9.4	**Chain A**: VAL3 GLU5 ALA6 TRP1 VAL3 GLU5 ALA6 TRP1 VAL3 GLU5 ALA6 TRP1 VAL3 GLU5 ALA6 TRP1 VAL3 GLU5 ALA6 TRP1 VAL3 GLU5 ALA6 TRP1 VAL3 GLU5 ALA6 TRP1 VAL3 GLU5 ALA6 VAL3 GLU5 ALA6 ASP8 TRP1 VAL3 GLU5 ALA6	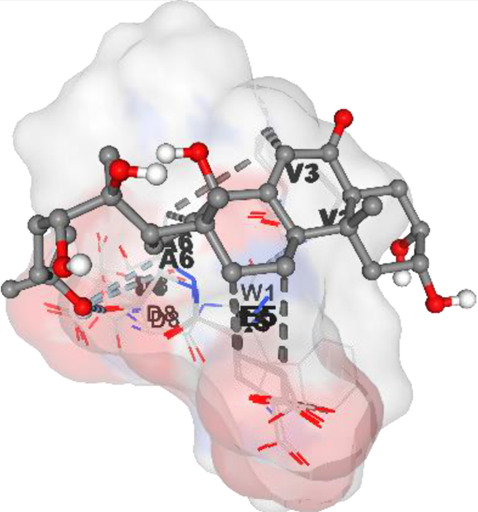	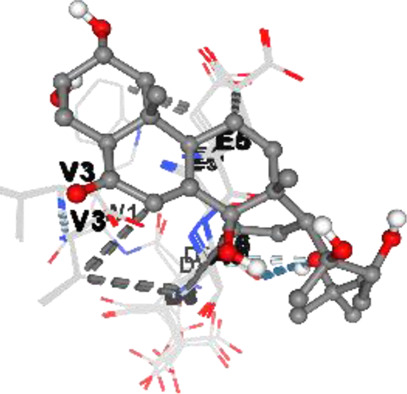
**PD-L1/**Ecdysterone	−9.4	**Chain A:** GLN139 ILE141 THR203 ASN204 GLU223 LEU224 VAL225 ILE226 PRO227 GLU228 LEU229 PRO230 **Chain B:** TYR32 GLY33 SER34 ASP103 LYS136 ASN138 GLN156 ALA157 GLU158 THR182 SER184 LYS185 ARG186 GLU187 LEU190 ASN192 THR194	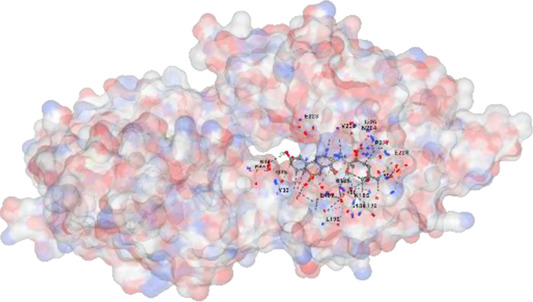	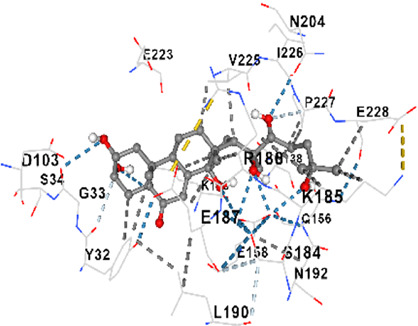
**DNMT-1/**Inokosterone	10.0	**Chain B**: MET651 ARG654 CYS656 GLY657 VAL658 VAL699 LYS700 GLU701 ALA702 ASP703 ASP704 ASP705 GLU706 GLU707 TYR983 ILE984 LYS985 GLY986 SER987 PHE1148 SER1149 GLY1150 GLU1171 MET1172 TRP1173 ALA1176 GLY1226 PRO1227 PRO1228 CYS1229 GLN1230 GLY1231 PHE1232 SER1249 LEU1250 VAL1251 GLU1269 ASN1270 VAL1271 ARG1272 THR1312 ARG1313 ARG1314 ARG1315 ARG1340 ALA1341 PHE1524 PHE1525 SER1526 THR1527 THR1528 VAL1529 THR1530 ASN1531 GLU1533 GLY1536 LYS1537 GLN1538 ARG1540 ARG1576 GLY1579 ASN1580 ALA1581 VAL1582	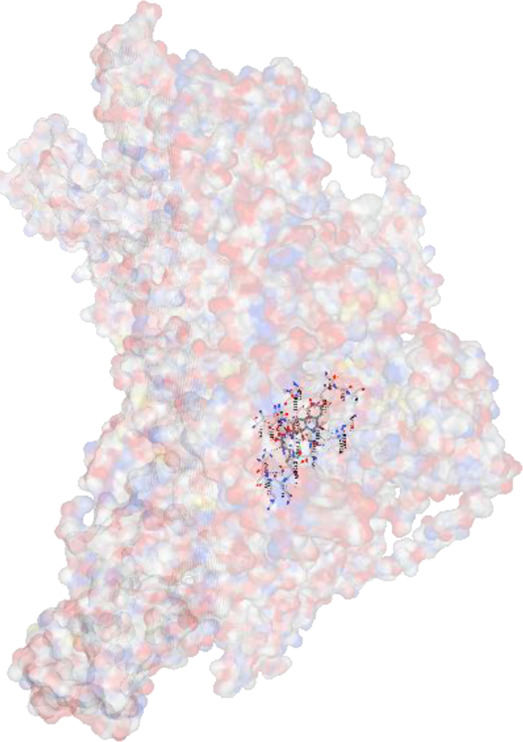	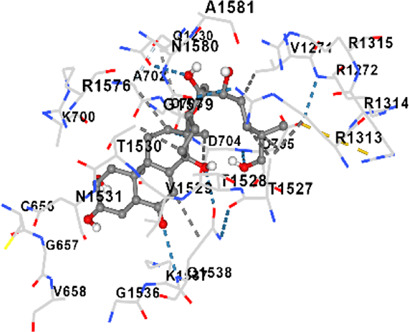
**Dicer/**Inokosterone	−9.6	**Chain A**: ASP340 GLU365 GLU369 ARG372 GLU374 ALA375 TYR378 SER399 LYS400 THR401 ASP404 GLU407 GLU435 HIS436 SER478 VAL479 PHE480 CYS481 SER482 SER483 ARG484 SER486 **Chain B**: GLN333 GLU336 LEU337 LEU338 ASP340 ALA341 MET368 GLU369 ARG372 ASN373 GLU374 ALA375 ASN377 TYR378 LYS398 SER399 LYS400 ASP404 GLU407 CYS481 SER482 SER483 SER486 LYS657 GLU673 THR677 ARG680 GLN681	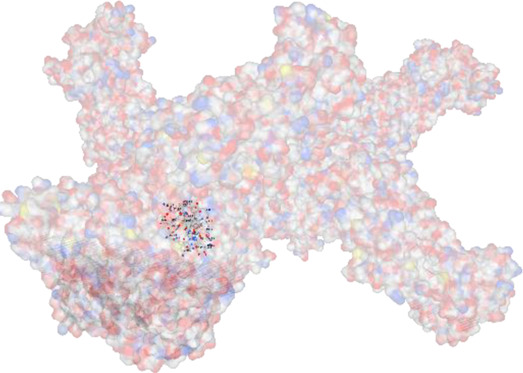	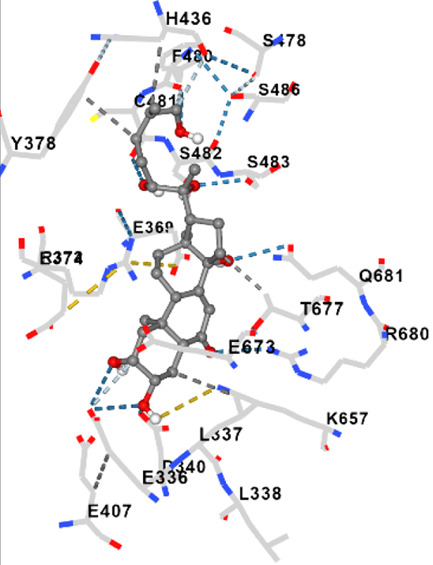
**PD-1/**Inokosterone	−9.2	**Chain A**: TRP1 VAL3 GLU5 ALA6 ASP8 TRP1 VAL3 GLU5 ALA6 TRP1 VAL3 GLU5 ALA6 TRP1 VAL3 GLU5 ALA6 TRP1 VAL3 GLU5 ALA6 TRP1 VAL3 GLU5 ALA6 TRP1 VAL3 GLU5 ALA6 ASP8 TRP1 VAL3 GLU5 ALA6 VAL3 GLU5 ALA6 ASP8 TRP1 VAL3 GLU5 ALA6 ASP8	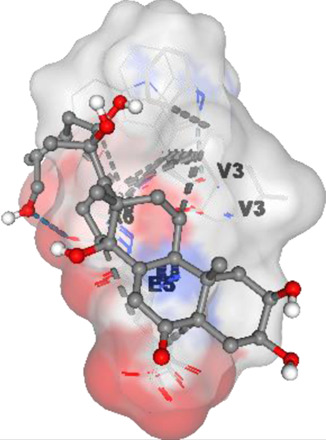	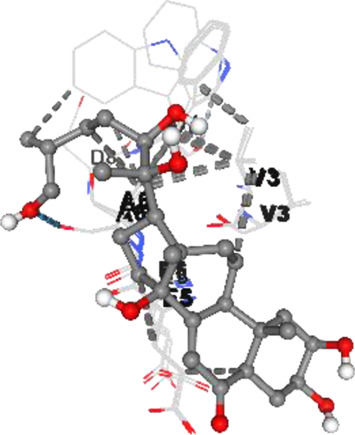
**PD-L1/**Inokosterone	−9.0	**Chain A:** GLN139 ILE141 THR203 ASN204 GLU223 LEU224 VAL225 ILE226 PRO227 GLU228 LEU229 **Chain B:** GLU31 TYR32 GLY33 SER34 ASP103 LYS105 ALA132 PRO133 ASN135 LYS136 ASN138 GLN156 ALA157 GLU158 GLY159 THR182 ASN183 SER184 LYS185 ARG186 GLU187 LEU190 ASN192 THR194	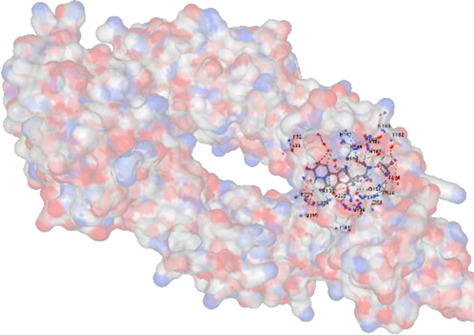	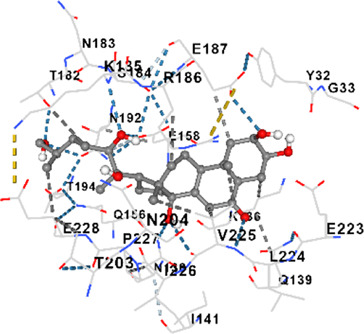

## 4 Expanding prostate cancer therapeutics with herbal medicine

### 4.1 Current landscape of present prostate cancer therapy

Prostate cancer remains one of the most prevalent cancers affecting men worldwide. Despite advances in androgen-targeting therapies, resistance mechanisms like CRPC continue to challenge treatment outcomes. Androgen-targeting therapies often create a counterproductive cycle by promoting adaptive mechanisms within cancer cells, making them more resilient over time. Given the variability in patient responses to hormone-targeting therapies, exploring alternative therapeutic strategies is essential. Targeting multiple pathways, beyond just the androgen receptor, could provide more sustainable and effective outcomes tailored to individual patient profiles. Elderly cancer patients, in particular, face limitations due to the significant risks associated with conventional therapies (Kantarjian et al., 2012). Thus, it is crucial to explore alternative compounds, particularly those under investigation for prostate cancer and CRPC, to develop promising options for future anticancer therapies.

### 4.2 Challenges and limitations in targeting DNMT1 in prostate cancer

While DNMT1 remains a promising therapeutic strategy in prostate cancer, its clinical application faces a significant challenges and limitations. Currently two DNMT inhibitors (DNMTi), 5-azacytidine (azacitidine) and 5-aza-2′-deoxycytidine (decitabine) have been approved by Food and Drug Administration (FDA) and European Medicines Agency (EMA) for the treatment of myelodysplastic syndromes (MDS), acute myeloid leukemia (AML), and chronic myelomonocytic leukemia (CMML) (Constantinides et al., 1978; Jones et al., 2019). Despite their therapeutic potential, these inhibitors pose several critical challenges. DNMT inhibitors cause widespread DNA hypomethylation, which, while reactivating tumor suppressor genes, can inadvertently activate oncogenes, potentially promoting tumorigenesis rather than suppressing it (Storebjerg et al., 2018). Intertumoral heterogeneity (ITH), characterized by diverse CpG methylation patterns, disrupts key cellular processes, including epigenetic modification (Portela and Esteller, 2010). Both 5-azacytidine and decitabine can induce DNA double-strand breaks, which may lead to genomic instability and increased toxicity in non-cancerous tissues, raising concerns about long-term safety (Paczkowski et al., 2021). DNMT inhibitors can lead to DNA double-strand breaks, potentially causing toxicity in non-cancerous tissues and resulting in adverse side effects lead to off-target effects and toxicity (Santi et al., 1984; Chen et al., 1991).

### 4.3 Limitations and challenges in targeting dicer

Dicer’s function in prostate cancer is context-dependent, where its expression may either promote or suppress tumor progression based on the disease stage and microenvironment. For example, elevated Dicer expression is linked to tumor aggression in early stages, while its dysfunction in advanced stages exacerbates treatment resistance (Bian et al., 2014). This dual role complicates therapeutic targeting strategies.

Hypoxic conditions in the tumor microenvironment exacerbate Dicer dysfunction, leading to downregulation of critical miRNAs like miR-124 and miR-144, which are associated with autophagy and treatment resistance. Addressing these hypoxia-driven effects is essential for successful therapeutic targeting (Gu et al., 2016). While effective delivery of Dicer-targeting agents to prostate cancer tissues remains a challenge. Current approaches often result in non-specific delivery, potentially affecting normal tissues and causing toxicity.

### 4.4 The promise and challenges of immunotherapy

The lymphatic system, a key player in maintaining fluid balance, lipid absorption, and immune regulation, has emerged as an important target in cancer therapy. Disruptions in lymphangiogenesis are linked to poor prognosis in cancer patients, including those with prostate cancer (Hu et al., 2024). While immunotherapy, particularly PD-1/PD-L1 inhibitors, has revolutionized cancer treatment, it is not without limitations. Immune-related adverse events (irAEs) pose significant challenges, as overstimulation of the immune system can lead to damage to healthy tissues, affecting the skin, liver, lungs, endocrine glands, and gastrointestinal tract (Baxi et al., 2018). These adverse effects can range from mild to life-threatening conditions, underscoring the need for precise biomarkers to predict immune responses and minimize risks.

While immunotherapy offers transformative potential in prostate cancer treatment, it is accompanied by significant challenges, including immune-related adverse events, tumor heterogeneity, and the complexity of PD-L1 regulation. The need for validated biomarkers, context-specific protein validation, and more refined therapeutic strategies is clear. Addressing these limitations through interdisciplinary research and precision medicine approaches will be pivotal in maximizing the clinical benefits of immunotherapy for prostate cancer patients (Tang et al., 2022). Further studies are needed to validate PD-L1 protein expression across different tumor microenvironments and prostate cancer subtypes. Investigations should also focus on post-transcriptional regulation mechanisms and protein stability factors influencing PD-L1 activity. Notably, combining PD-1/PD-L1 inhibitors with other therapeutic strategies (e.g., chemotherapy, targeted therapies, or natural compounds) may enhance therapeutic efficacy while reducing adverse effects.

### 4.5 Integrating herbal medicine into prostate cancer therapy: opportunities and challenges

Herbal medicines have gained increasing recognition for their potential to complement conventional prostate cancer therapies. Clinical trials have demonstrated that natural agents such as Modified Citrus Pectin (Keizman et al., 2023), pomegranate extract (Paller et al., 2013; Jarrard et al., 2021), and sulforaphane-rich broccoli sprouts (Alumkal et al., 2015) can stabilize PSA levels and exert anti-tumor effects. Additionally, compounds like muscadine grape skin extract (Paller et al., 2018) and saw palmetto (Wyatt et al., 2016) have shown promise in managing symptoms associated with prostate cancer. Despite these promising outcomes, significant challenges remain. Many herbal compounds have poor bioavailability, limiting their therapeutic efficacy in clinical settings (Patel et al., 2022). Inconsistent standardization of herbal formulations and variability in active compound concentrations pose challenges for reproducibility and dose optimization (Jenča et al., 2024). The molecular mechanisms by which herbal compounds exert their effects on prostate cancer pathways remain partially understood(Sharma et al., 2023). Herbal medicines often face regulatory hurdles due to insufficient clinical trial data supporting their safety and efficacy (Youn et al., 2023). Limited studies exist on the synergistic effects of herbal compounds with standard therapies, and their interactions with conventional drugs remain underexplored. To address these challenges, future research should focus on improving formulation strategies, employing nanotechnology-based delivery systems, and conducting well-designed clinical trials to validate the safety and efficacy of herbal compounds in prostate cancer therapy.

### 4.6 A holistic and multi-targeted approach to prostate cancer treatment

Integrating herbal medicines into standard oncology practices offers a holistic approach to prostate cancer care, focusing on both therapeutic efficacy and patient wellbeing. Conventional therapies, while effective, are often associated with high toxicity profiles and financial burdens exceeding $30,000 per month (Raudenska et al., 2019). In contrast, herbal medicines, with their long history of safe use in traditional practices, provide a cost-effective and complementary strategy for prostate cancer management (Tayeb et al., 2024). Theirs efficacy and safety have been demonstrated over centuries, successfully complementing modern medical practices (Chen Q et al., 2023). However, this approach is not without challenges. Different patient populations may exhibit heterogeneous responses to herbal interventions. Many studies remain preclinical, and robust Phase III trials are scarce. Notably, herbal therapies often require long-term administration, posing adherence challenges. To fully harness the potential of herbal medicines, it is essential to standardize formulations, optimize delivery mechanisms, and integrate them into personalized treatment regimens based on individual patient profiles.

### 4.7 A new avenue in prostate cancer therapy with BK002

BK002, an innovative herbal formulation, has emerged as a potential game-changer in prostate cancer therapy due to its multi-targeted mechanisms of action. Enriched with a diverse array of bioactive compounds, including flavonoids, terpenoids, and steroids, BK002 demonstrates anti-cancer, immune-modulating, and anti-inflammatory properties, positioning it as a compelling candidate for integrative cancer treatment strategies. Our studies have highlighted BK002s synergistic potential when combined with conventional chemotherapeutics and herbal decoctions, particularly in addressing critical biomarkers such as DNMT1, Dicer, PD-1, and PD-L1. These biomarkers are pivotal in regulating key pathways associated with epigenetic modifications, immune evasion, and cellular proliferation in prostate cancer, including CRPC. The integration of natural compounds such as ecdysterone, inokosterone, and 20-hydroxyecdysone within BK002 adds further therapeutic value. These compounds have shown promise in modulating prostate cancer pathways, potentially enhancing therapeutic efficacy while simultaneously reducing adverse effects commonly associated with standard therapies. However, significant challenges remain. The precise biochemical pathways through which BK002 exerts its anticancer effects require further elucidation. The potential interactions with existing chemotherapeutic agents need to be thoroughly examined to avoid unforeseen complications. While preclinical data are encouraging, the absence of large-scale clinical trials hampers the transition of BK002 from bench to bedside. Thus, future research directions should prioritize. Large-scale, well-designed trials to validate BK002s safety, efficacy, and optimal dosing protocols. In-depth exploration of BK002s molecular interactions with prostate cancer pathways to uncover novel therapeutic targets. Evaluation of BK002 in combination therapies to enhance therapeutic outcomes and reduce side effects. By bridging these knowledge gaps, BK002 has the potential to refine existing treatment paradigms, offering a holistic and personalized therapeutic approach to prostate cancer management. Continued interdisciplinary research will be essential to fully unlock the therapeutic promise of BK002 and establish its role as a standard adjunct therapy in prostate cancer care.

## 5 Conclusion

This study highlights the significant therapeutic potential of BK002, which contains ecdysterone, inokosterone, and 20-hydroxyecdysone, in the treatment of prostate cancer. These phytochemicals represent a promising complementary approach to conventional therapies, especially in the context of CRPC, where therapeutic resistance poses a substantial challenge. By targeting key biomarkers such as DNMT1, Dicer, PD-1, and PD-L1, these compounds have the potential to enhance the efficacy of existing treatment regimens while minimizing adverse effects. Moving forward, interdisciplinary research is crucial to further elucidate the molecular interactions and mechanisms of action of these compounds. A deeper understanding of these interactions could lead to the development of more effective combination therapies that harness the synergistic potential of herbal medicine alongside conventional cancer treatments. This integrative approach marks a significant shift towards more sustainable, patient-centered cancer care, with the potential for improved outcomes in prostate cancer treatment and beyond. By advancing the scientific foundation for incorporating herbal medicines into oncology, this research aims to transform prostate cancer treatment. Moving away from the traditional one-size-fits-all approach, these personalized, holistic strategies emphasize both treatment efficacy and patient wellbeing. Further exploration of these natural compounds could lead to the creation of novel therapeutic protocols that enhance the overall quality of cancer care, offering a more comprehensive and sustainable path forward in the fight against prostate cancer.
